# Diversity, climatic correlations, and biocontrol prospects of seed-borne fungal endophytes in Egyptian maize

**DOI:** 10.1038/s41598-026-41567-3

**Published:** 2026-03-26

**Authors:** Khadiga A. Hasan, Hoda M. Soliman, Khalid M. Ghoneem, Yasser M. Shabana

**Affiliations:** 1 Botany Department, Faculty of Science, Mansoura University, Mansoura, 35516 Egypt; 2 Seed Pathology Research Department, Plant Pathology Research Institute, Agricultural Research Center, Giza, 12619 Egypt; 3 Department of Plant Pathology, Faculty of Agriculture, Mansoura University, Mansoura, 35516 Egypt

**Keywords:** Maize, Seed-borne endophytes, Fungal diversity, Climatic factors, *Fusarium verticillioides*, *Trichoderma longibrachiatum*, Biocontrol, Egypt, Biotechnology, Ecology, Ecology, Microbiology, Plant sciences

## Abstract

Maize (*Zea mays* L.) is the world’s third most important cereal crop, valued for its roles in human food, animal feed, industrial products, and biofuels. Its seeds harbor diverse endophytic fungi that can affect seed quality, plant health, and resilience. Given the vertical transmission of seed microbiota, this study investigated the diversity of seed-borne fungal endophytes in Egyptian maize cultivars across 18 governorates and assessed their associations with regional climate. In addition, the study evaluated the antagonistic activity of native *Trichoderma* isolates as potential biocontrol agents against *Fusarium verticillioides*, a major seed-borne pathogen threatening maize production. A total of 34 endophytic fungal species from 23 genera were identified. *Aspergillus niger*, *Penicillium* spp., *A. flavus*, and *F. verticillioides* were the most prevalent, with *A. niger* occurring in 97.2% of sites and *F. verticillioides* in 89.6%. Relative abundances were highest for *Penicillium* spp. (20.08%), *A. niger* (18.15%), and *F. verticillioides* (14.93%). Diversity metrics varied regionally, with species richness ranging from 8 to 22 and Shannon diversity indices (H) from 1.02 to 2.46. Canonical correspondence analysis revealed that temperature, solar radiation, and humidity collectively explained 63.6% of fungal community variation. *Trichoderma longibrachiatum* (T14) demonstrated the strongest antagonistic effect, inhibiting *F. verticillioides* growth by 74.03% in vitro and exhibiting pronounced mycoparasitic features. This study highlights the dominance of a core group of seed-borne endophytes in Egyptian maize and underscores the significant role of climate in structuring fungal communities. However, as this study was based on single-season, culture-dependent data with limited molecular validation, further multi-seasonal and molecular-based investigations are needed to confirm these patterns and fully characterize the maize seed mycobiome. Notably, *T. longibrachiatum* demonstrated strong biocontrol potential against *F. verticillioides*, offering promise as a native, eco-friendly biocontrol agent for maize production systems in semi-arid environments.

**Supplementary Information:**

The online version contains supplementary material available at 10.1038/s41598-026-41567-3.

## Introduction

Maize (*Zea mays* L.) is the third most important cereal crop globally, following rice and wheat, and plays a critical role in global food security, animal feed systems, feedstock for several industrial products, and a source of biofuels^1–3^. In Egypt, maize is cultivated across nearly 913,000 hectares, with an estimated annual production of 6.9 million tonnes^4^. This cultivation spans diverse agro-climatic regions, from the Nile Valley to the Delta and Upper Egypt, offering a unique opportunity to investigate how local environmental conditions shape seed-associated fungal communities^5^.

Despite its global importance, maize remains highly vulnerable to a wide range of fungal pathogens, many of which are seed-borne and capable of reducing grain quality, yield, and viability. Among these, *F. verticillioides* stands out for its destructive capacity, it causes seed rot, impairs germination, and induces ear and stalk infections that result in fumonisin mycotoxin accumulation, posing serious risks to both human and animal health^6^. Recent metabolomic and field studies have revealed that maize kernels infected by this fungus can accumulate high levels of FB_1_ and FB_2_ toxins while remaining symptom-free, making contamination difficult to detect^7^. Furthermore, *F. verticillioides* often dominates as the major toxigenic species in maize-growing regions, with its persistence in seeds and under storage conditions further intensifying the problem^8^. These factors highlight the urgent need for effective and sustainable management strategies.

Historically, the management of *F. verticillioides* has relied heavily on chemical fungicides. Systemic formulations such as tebuconazole + trifloxystrobin and prochloraz have been shown to suppress fungal mycelial growth by over 80% under laboratory conditions^9^. Similarly, the volatile compound 1-octyn-3-ol completely inhibited fungal growth and fumonisin B_1_ production during grain storage^10^. However, despite their short-term efficacy, the extensive use of synthetic fungicides raises environmental and health concerns, including residual toxicity, soil contamination, and the emergence of resistant strains^11^. These drawbacks have prompted a global shift toward eco-friendly and biologically based alternatives^12^.

In this context, biological control agents have gained increasing attention as environmentally safe alternatives. Among these, endophytic fungi are particularly promising due to their ability to colonize internal plant tissues without causing harm, while simultaneously enhancing plant growth, stress tolerance, and disease resistance^13–15^. The dual role of endophytes in promoting plant health and enhancing host defense makes them valuable components of sustainable agricultural strategies^16–21^.

Previous studies have provided compelling evidence for the biocontrol potential of maize-associated endophytic fungi against *Fusarium* pathogens. For example, Li et al.^8^ reported that a maize-derived endophyte exhibited dual antagonism against *F. verticillioides*, effectively inhibiting its growth and reducing fumonisin accumulation. Likewise, Mirsam et al.^22^ demonstrated that indigenous maize endophytes suppressed the pathogen by over 50% in vitro and improved seed germination. Together, these findings underscore that maize-associated endophytes not only suppress diseases but also enhance plant vigor and resilience, offering an ecologically compatible approach for integrated pathogen management.

Building on these promising insights, it remains essential to further understand how seed-borne endophytic fungi adapt and function under different environmental conditions, particularly within arid and semi-arid ecosystems.

Although previous research has highlighted the potential of endophytic fungi as effective biocontrol agents, a comprehensive understanding of their diversity and ecological adaptation in arid regions remains limited. Most existing research has focused on endophytic fungi associated with maize roots and leaves, offering valuable insights into their roles in plant growth promotion and disease resistance^23–26^. However, much less is known about seed-borne endophytes and their adaptive responses across contrasting environmental conditions.

The structure and composition of endophytic fungal communities are influenced by multiple environmental factors, including temperature, relative humidity, precipitation, and solar radiation^27–32^. These variables not only affect fungal abundance and distribution but also drive regional variations in community diversity and functional traits^33–37^. Among them, precipitation plays a particularly important dual role, facilitating the dispersal of fungal propagules while simultaneously shaping community composition through microclimatic effects^38,39^.

Within this ecological framework, species belonging to the genus *Trichoderma* have gained growing attention for their ability to suppress plant pathogens through multiple mechanisms, including competition, antibiosis, and mycoparasitism. Several *Trichoderma* species have been recognized as effective biocontrol agents against *Fusarium* pathogens^40,41^. However, most previous work has focused on soil-borne or foliar applications, while the potential of seed-borne *Trichoderma* isolates, particularly native strains adapted to local agroecological conditions, remains largely unexplored.

Therefore, this study was designed to address these knowledge gaps by: (i) characterizing the diversity and distribution of seed-borne endophytic fungi in maize seeds collected from 18 agroecologically diverse Egyptian governorates; (ii) analyzing the influence of regional climatic variables on the structure of these fungal communities; and (iii) evaluating the antagonistic potential of selected native *Trichoderma* isolates, particularly *Trichoderma longibrachiatum*, as prospective biocontrol agents against *F. verticillioides*, a key seed-borne pathogen of maize. This integrated approach aims to combine ecological characterization with preliminary biocontrol screening to support future endophyte-based disease management strategies in semi-arid maize production systems.

## Results

### Morphological characterization of endophytic fungi

Microscopic and cultural examinations revealed pronounced morphological diversity among the isolated endophytic fungi. When cultivated on potato dextrose agar (PDA), the isolates exhibited distinct variations in colony texture, pigmentation, and growth rate. Genera such as *Aspergillus* and *Penicillium* typically formed dense, pigmented colonies with abundant sporulation, whereas *Fusarium* species developed cottony, aerial mycelia with pink to violet pigmentation. *Trichoderma* isolates were readily distinguished by their rapid radial growth and the progressive -colour transition from white to green as conidia matured.

**Fig. 1:**
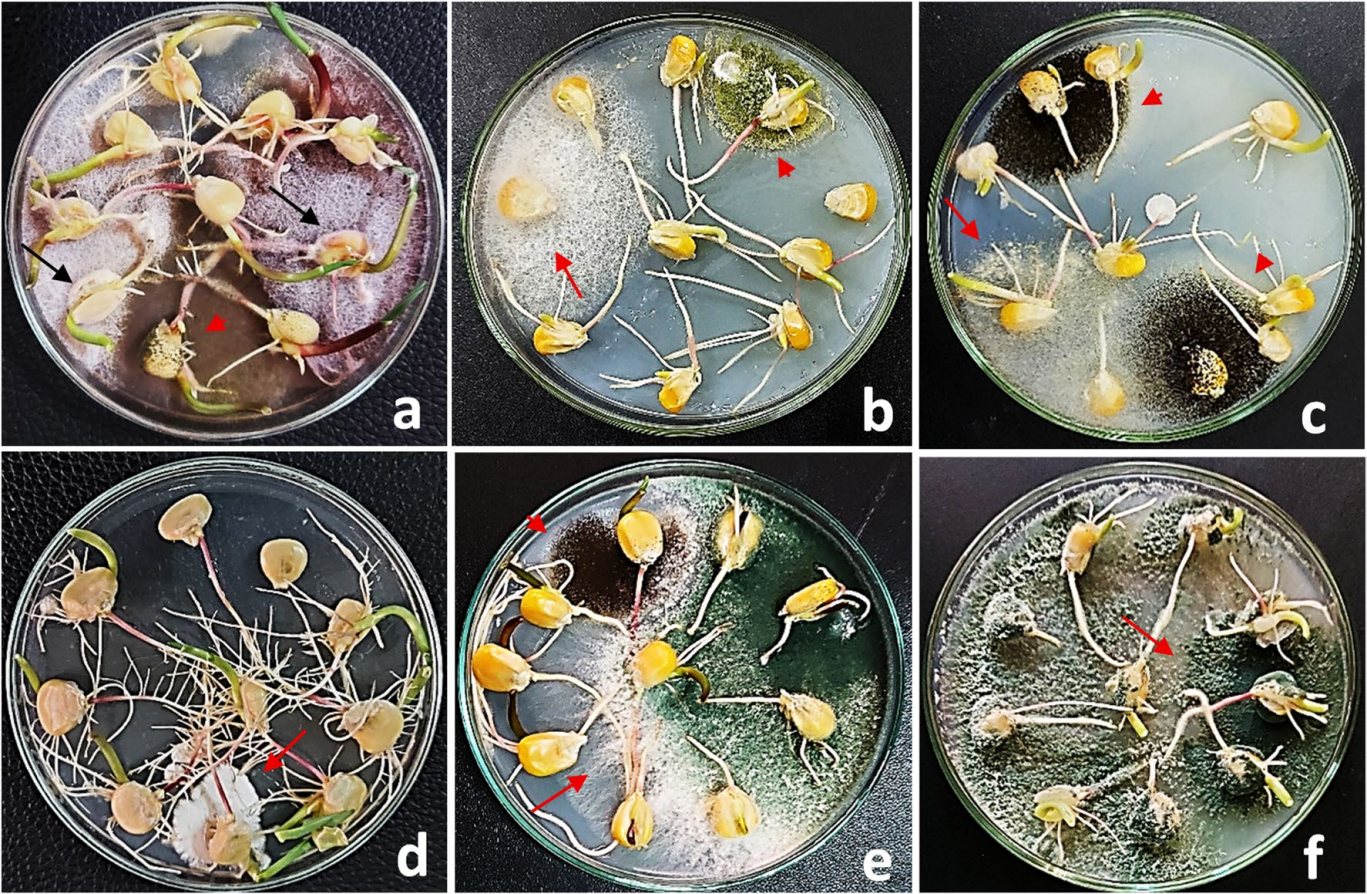
Habit characters of endophytic seed-borne mycobiomes on maize seeds showing: (**a**) whitish to purplish-white, cottony and profuse mycelium of *Fusarium verticillioides* covering the seed (arrow), and fast-growing, light to dark brown mycelium of *Chaetomium* spp. with submerged, loose brown perithecia covering the entire seed (arrowhead); (**b**) yellow-green conidial heads of *Aspergillus flavus* on maize seed (arrowhead); (**c**) whitish to buff-brown, smooth, cottony mycelium of *F. incarnatum* (arrow) and black powdery conidial heads of *A. niger* (arrowhead) covering the seeds; (**d**) slimy colonies of *Aureobasidium pullulans* covering the seeds (arrow); and (**e**,**f**) yellow-green, fast-spreading aerial mycelium of *Trichoderma* spp. confined below and around the seeds (arrows). Photographs were taken after 4 days of incubation using the agar plate (AP) method.

**Fig. 2:**
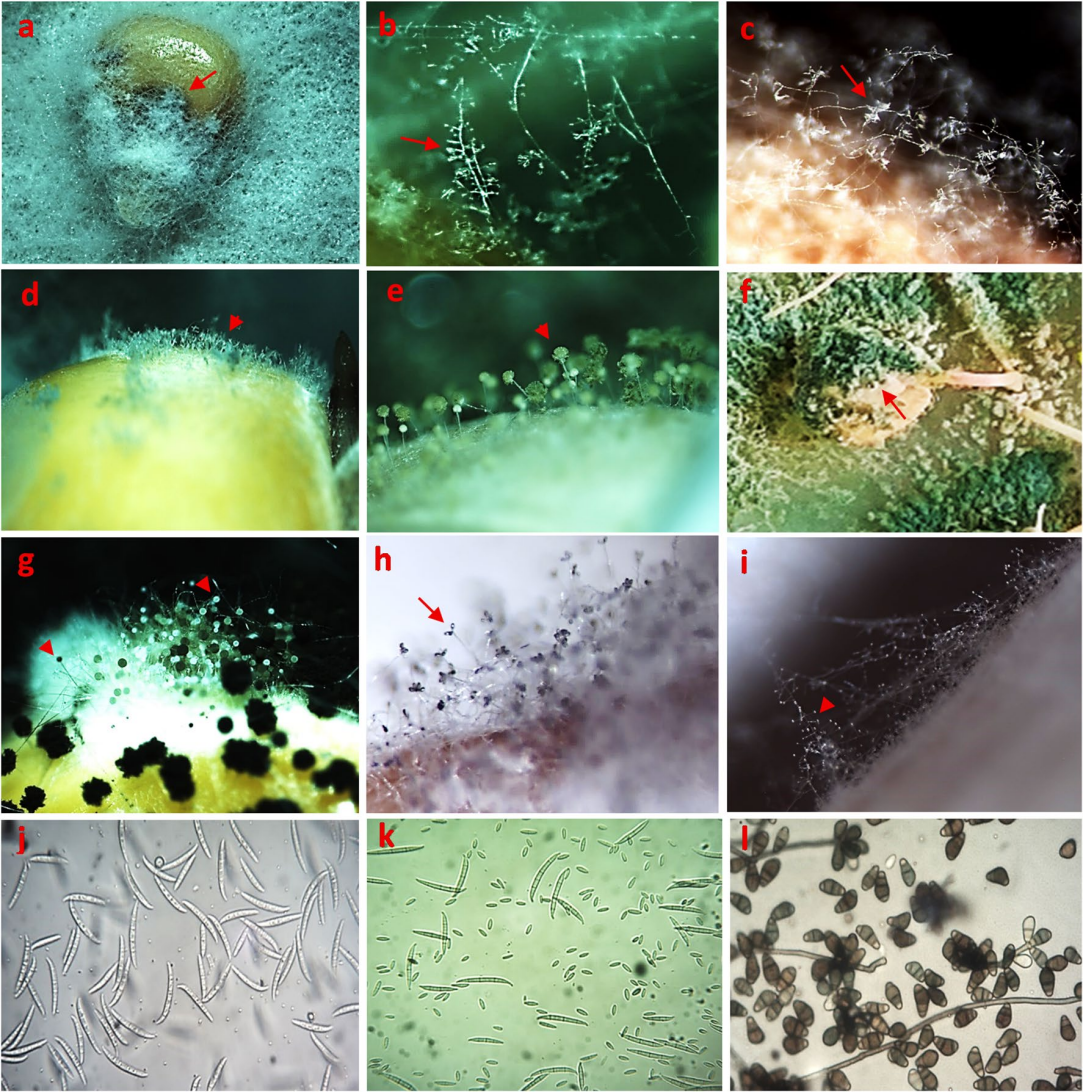
Stereoscopic and microscopic micrographs of dominant endophytic fungi isolated from maize seeds showing: (**a**, ×64; **b**, ×120) whitish, powdery mycelial growth and conidiophores bearing chains of microconidia and spore heads of *Fusarium verticillioides* (arrows); (**c**, ×64) branched conidiophores bearing conidia of *F. incarnatum* (arrow); (**d**, ×64) abundant mycelial growth and spore heads of *Penicillium* spp. (arrowhead); (**e**, ×120) *Aspergillus flavus* spore heads (arrowhead, (**f**, ×64) yellowish-green, fluffy to velvety conidiophores and conidia of *Trichoderma* spp. covering maize seeds (arrow); (**g**, ×64) black powdery mycelium and spore heads of *A. niger* (arrowheads); (**h**, ×64) dense, dark brown, velvety conidiophores and conidia of *Curvularia lunata* (arrowhead); (**i**, ×64) white mycelium bearing conidiophores ending with conidial droplets of *Cephalosporium acremonium* conidia (arrowhead; (**j**, ×1000) typical macroconidia and chlamydospores of *F. incarnatum*; (**k**, ×1000) micro- and macroconidia of *F. verticillioides*; and (**l**, ×1000) smooth-walled, dark conidia and conidiophore of *C. lunata*.

At the microscopic level, the isolates displayed diagnostic features in conidiophore branching, phialide arrangement, and conidial morphology, consistent with their respective genera. Representative colony and microscopic characteristics are illustrated in Figs. 1 and 2, and key diagnostic traits are summarized in Supplementary Table S1.

Collectively, these morphological observations confirm the distinct taxonomic identity of the isolates and provide a reliable foundation for subsequent analyses of ecological distribution and antagonistic potential.

### Occurrence and distribution of maize seed-borne endophytic mycoflora

Building on these morphological characterizations, the seed-borne endophytic mycobiota of maize were subsequently analyzed to determine their occurrence and distribution across Egypt’s major maize-growing governorates during the 2021 cropping season.

A total of 144 maize grains samples were collected from eighteen maize-growing governorates (8 site per governorate). These samples included thirteen hybrids of Egyptian maize cultivars. Among them SC-168, SC-178, SC P3433, TWC-360, and TWC-368 are yellow maize hybrids, while SC-30 K8, SC-30 K9, SC-132, SC-2030, SC-2031, SC-30P74, TWC-321, and TWC-324 represent white maize hybrids.

**Fig. 3:**
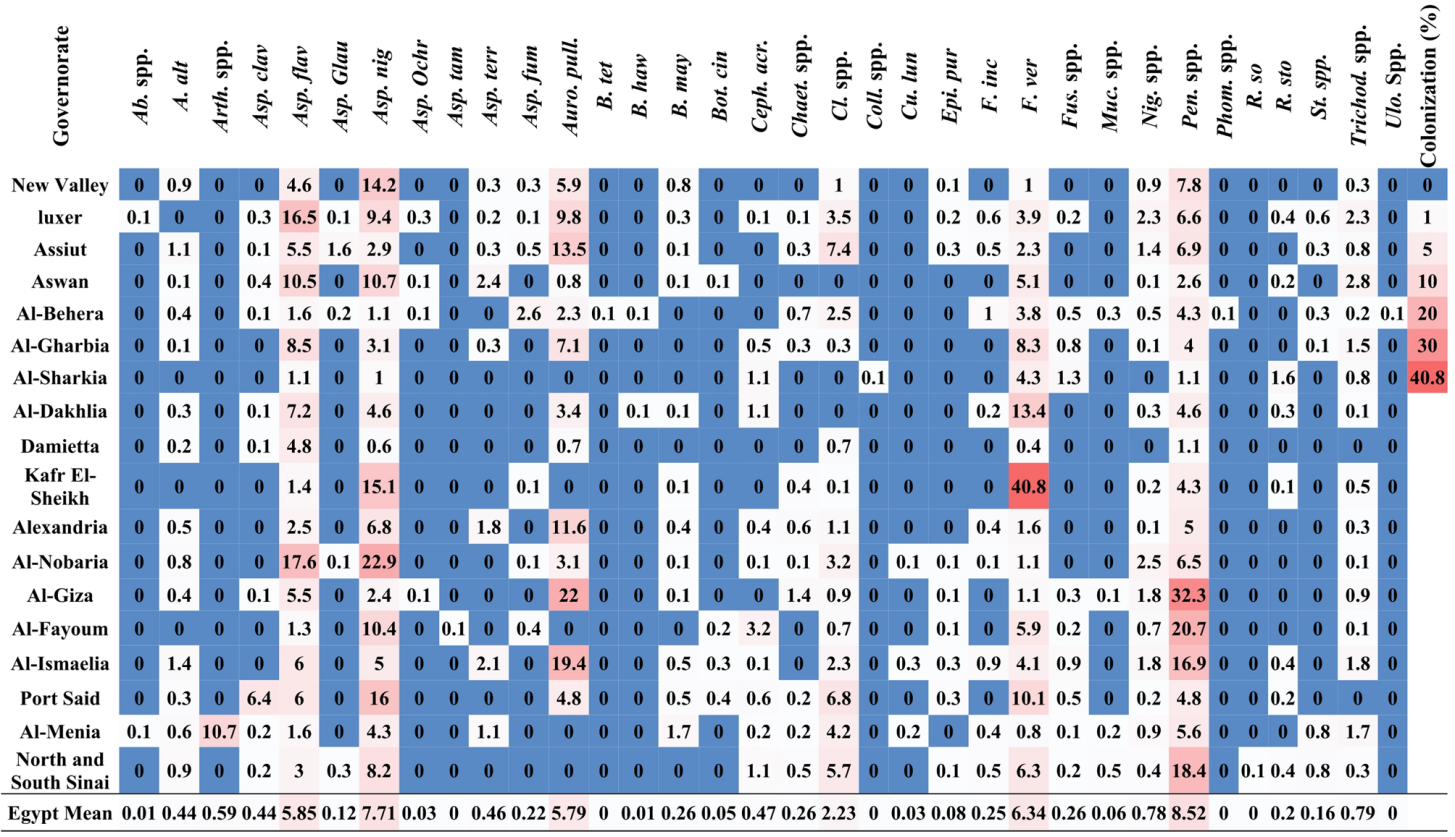
Heatmap showing colonization percentages of endophytic fungi isolated from maize seeds across Egypt’s 18 maize-growing governorates. Each cell represents the mean colonization percentage of a fungal species within a governorate, averaged across all sampling sites. The gradient scale ranges from 0% (blue) to 40.8% (red). *Ab*. spp. (*Absidia* spp.), *(A) alt*. (*Alternaria alternata*), *Arth.* spp. (*Arthrobotrys* spp.), *Asp. cla*. (*Aspergillus clavatus*), *Asp. fla.* (*A. flavus*), *Asp. fum*. (*A. fumigatus*), *Asp. gla*. (*A. glaucus*), *Asp. nig*. (*A. niger*), *Asp. och*. (*A. ochraceus*), *Asp. tam.* (*A. tamarii*), *Asp. terr.* (*A. terreus*), *Auro. pul.* (*Aureobasidium pullulans*), *(B) haw*. (*Bipolaris hawaiiensis*), *B. may.* (*B. maydis*), *B. tet.* (*B. tetramera*), *Bot. cin.* (*Botrytis cinerea*), *Ceph. acr.* (*Cephalosporium acremonium*), *Chaet.* spp. (*Chaetomium* spp.), *Cl.* spp. (*Cladosporium* spp.), *Coll.* spp. (*Colletotrichum* spp.), *Cu. lun*. (*Curvularia lunata*), *Epi. pur.* (*Epicoccum purpurascens*), *F. inc.* (*Fusarium incarnatum*), *Fus. spp.* (*Fusarium* spp.), *F. ver.* (*F. verticillioides*), Muc. spp. (*Mucor* spp.), Nig. spp. (*Nigrospora* spp.), Pen. spp. (*Penicillium* spp.), Phom. sp. (*Phoma* sp.), *R. so.* (*Rhizoctonia solani*), *R. sto.* (*Rhizopus stolonifer*), *St.* spp. (*Stemphylium* spp.), *Trichod. spp.* (*Trichoderma* spp.), and *Ulo*. spp. (*Ulocladium* spp.). The final row “Egypt” in the table represents the mean frequency (%) of each fungal species across all surveyed governorates, providing an overall profile of seed-borne fungal prevalence. This summary row was excluded from comparative analyses and visualization.

In total thirty-four endophytic fungal species belonging to twenty-three genera were detected in the maize grains. Colonization percentages were calculated based on the total number of colonies recovered per governorate (Table S2), ranged from 0.0% to 40.8% (Fig. 3), highlighting pronounced differences in fungal abundance among regions and taxa. The colonization rates of seed-borne fungi exhibited marked variation among Governorates and fungal species (Fig. 3). *Fusarium* spp. displayed the highest observed colonization rate, reaching 40.8% in one Governorate (in Kafr El Sheikh), indicating strong prevalence in certain regions followed by *Penicillium* spp. reached 32.25% colonization in Giza Governorate. Similarly, *A. niger* and *A. flavus* showed high colonization rates in multiple locations, with maximum percentages of 22.88% and 17.6%, respectively. In contrast, less frequent taxa such as *Ulocladium* spp., *Rhizoctonia solani*, and *Rhizopus* spp. had colonization rates below 1% in most Governorates.

The mean colonization percentages across all Governorates (“Egypt Mean”) further emphasized the predominance of *Penicillium* spp. (8.52%) and *A. niger* (7.71%), followed by *Fusarium* spp. **(**6.34%**)** and *A. flavus* (5.85%). The overall pattern highlights a small group of fungi consistently contributing to seed colonization across diverse ecological regions. The heatmap visualization underscores these trends, with warmer colors corresponding to higher colonization rates.

**Fig. 4:**
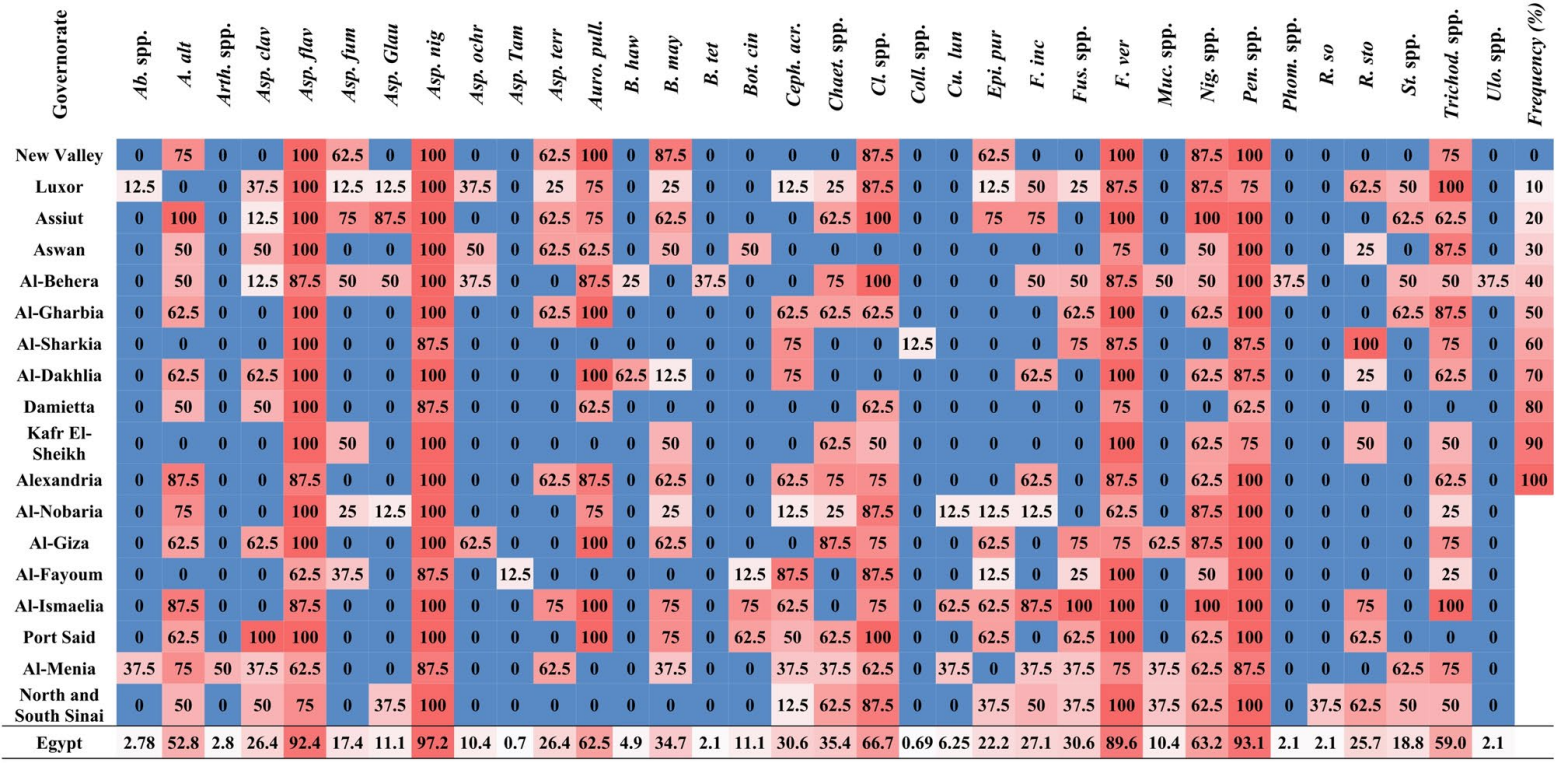
Heatmap showing the per-governorate frequencies of 34 endophytic fungal species associated with maize seeds collected from Egypt’s 18 maize-growing governorates. The frequency gradient ranges from 0% (blue) to 100% (red), as indicated on the scale bar. *Ab*. spp. (*Absidia* spp.), *(A) alt*. (*Alternaria alternata*), *Arth.* spp. (*Arthrobotrys* spp.), *Asp. cla*. (*Aspergillus clavatus*), *Asp. fla.* (*A. flavus*), *Asp. fum*. (*A. fumigatus*), *Asp. gla*. (*A. glaucus*), *Asp. nig*. (*A. niger*), *Asp. och*. (*A. ochraceus*), *Asp. tam.* (*A. tamarii*), *Asp. terr.* (*A. terreus*), *Auro. pul.* (*Aureobasidium pullulans*), *(B) haw*. (*Bipolaris hawaiiensis*), *B. may.* (*B. maydis*), *B. tet.* (*B. tetramera*), *Bot. cin.* (*Botrytis cinerea*), *Ceph. acr.* (*Cephalosporium acremonium*), *Chaet.* spp. (*Chaetomium* spp.), *Cl.* spp. (*Cladosporium* spp.), *Coll.* spp. (*Colletotrichum* spp.), *Cu. lun*. (*Curvularia lunata*), *Epi. pur.* (*Epicoccum purpurascens*), *F. inc.* (*Fusarium incarnatum*), *Fus. spp.* (*Fusarium* spp.), *F. ver.* (*F. verticillioides*), Muc. spp. (*Mucor* spp.), Nig. spp. (*Nigrospora* spp.), Pen. spp. (*Penicillium* spp.), Phom. sp. (*Phoma* sp.), *R. so.* (*Rhizoctonia solani*), *R. sto.* (*Rhizopus stolonifer*), *St.* spp. (*Stemphylium* spp.), *Trichod. spp.* (*Trichoderma* spp.), and *Ulo*. spp. (*Ulocladium* spp.). The final row labeled “Egypt” in the table represents the mean frequency (%) of each fungal species across all surveyed governorates, providing an overall profile of seed-borne fungal prevalence. This summary row was excluded from comparative analyses and visualization.

Frequencies per-governorate revealed marked variability in the distribution of endophytic fungi among regions (Fig. 4). In several governorates, notably Luxor and Al-Behera, common taxa such as *A. niger* and *Penicillium* spp. were detected in all eight sampled sites, corresponding to a per-governorate frequency of 100%.

### Endophytic mycobiome diversity in maize seeds

**Fig. 5:**
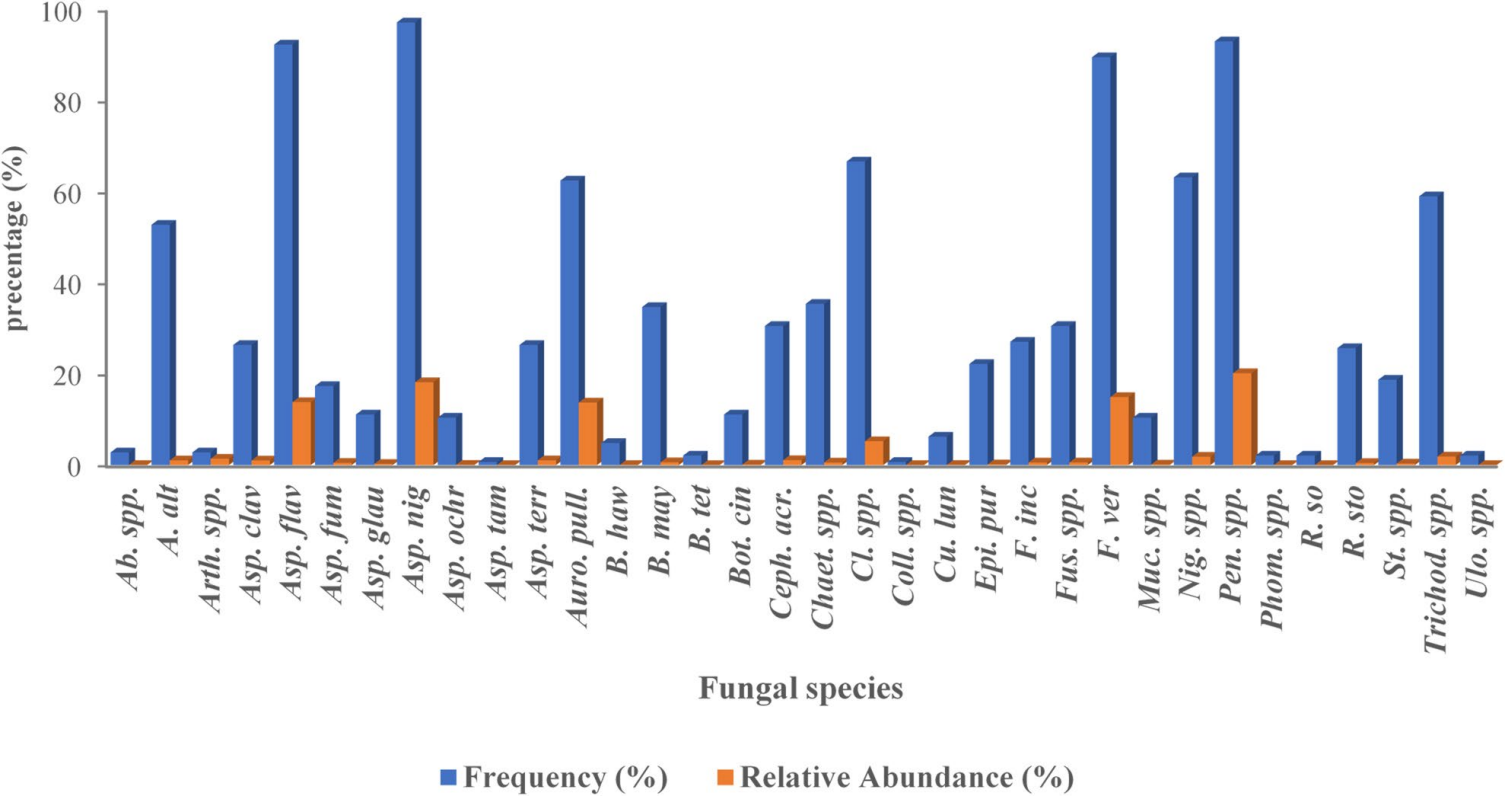
The Frequency (%) and Relative abundance (%) of endophytic fungi found in maize seeds across Egypt’s 18 governorates that grow maize. Values were calculated from data aggregated from all governorates. The blue bars show number of relative abundance (%), and the orange bars show the total frequency (%). *Ab*. spp. (*Absidia* spp.), *(A) alt*. (*Alternaria alternata*), *Arth.* spp. (*Arthrobotrys* spp.), *Asp. cla*. (*Aspergillus clavatus*), *Asp. fla.* (*A. flavus*), *Asp. fum*. (*A. fumigatus*), *Asp. gla*. (*A. glaucus*), *Asp. nig*. (*A. niger*), *Asp. och*. (*A. ochraceus*), *Asp. tam.* (*A. tamarii*), *Asp. terr.* (*A. terreus*), *Auro. pul.* (*Aureobasidium pullulans*), *(B) haw*. (*Bipolaris hawaiiensis*), *B. may.* (*B. maydis*), *B. tet.* (*B. tetramera*), *Bot. cin.* (*Botrytis cinerea*), *Ceph. acr.* (*Cephalosporium acremonium*), *Chaet.* spp. (*Chaetomium* spp.), *Cl.* spp. (*Cladosporium* spp.), *Coll.* spp. (*Colletotrichum* spp.), *Cu. lun*. (*Curvularia lunata*), *Epi. pur.* (*Epicoccum purpurascens*), *F. inc.* (*Fusarium incarnatum*), *Fus. spp.* (*Fusarium* spp.), *F. ver.* (*F. verticillioides*), Muc. spp. (*Mucor* spp.), Nig. spp. (*Nigrospora* spp.), Pen. spp. (*Penicillium* spp.), Phom. sp. (*Phoma* sp.), *R. so.* (*Rhizoctonia solani*), *R. sto.* (*Rhizopus stolonifer*), *St.* spp. (*Stemphylium* spp.), *Trichod. spp.* (*Trichoderma* spp.), and *Ulo*. spp. (*Ulocladium* spp.). Values were calculated from aggregated counts of colonies and the number of sites where each species was detected across Egypt.

Analysis of national frequency and relative abundance revealed that a small subset of endophytic fungi was both highly prevalent and dominant in maize seeds across Egypt. *A. niger* was the most widespread taxon, occurring in 97.22% of all sampled sites and accounting for 18.15% of total colony counts (relative abundance). *Penicillium* spp. and *A. flavus* were similarly frequent (93.06% and 92.36%, respectively) and exhibited high relative abundances (20.08% and 13.78%). *F. verticillioides* was also prominent, detected in 89.58% of sites and contributing 14.93% of relative abundance. In contrast, taxa such as *Aureobasidium pullulans* and *Cladosporium* spp. were detected in 62.50% and 66.67% of sites, respectively, yet showed markedly different relative abundances (13.63% and 5.26%) (Fig. 5). These patterns indicate that while certain species were consistently recovered across locations, their proportional representation in the total fungal community varied substantially.

The richness, and Shannon–Wiener diversity index (H) varied substantially among governorates. Richness values among governorates ranged from 8 species in Damietta to 22 species in both Luxor and Al-Behera while Shannon diversity indices spanned from 1.02 (Kafr El-Sheikh) to 2.46 (Al-Behera), reflecting marked differences in community evenness and complexity across regions. Notably, governorates such as Al-Behera, Luxor, and Assiut combined high richness with elevated Shannon indices, indicating diverse and relatively balanced fungal assemblages, whereas areas like Kafr El-Sheikh and Damietta exhibited lower diversity and richness overall.

**Fig. 6:**
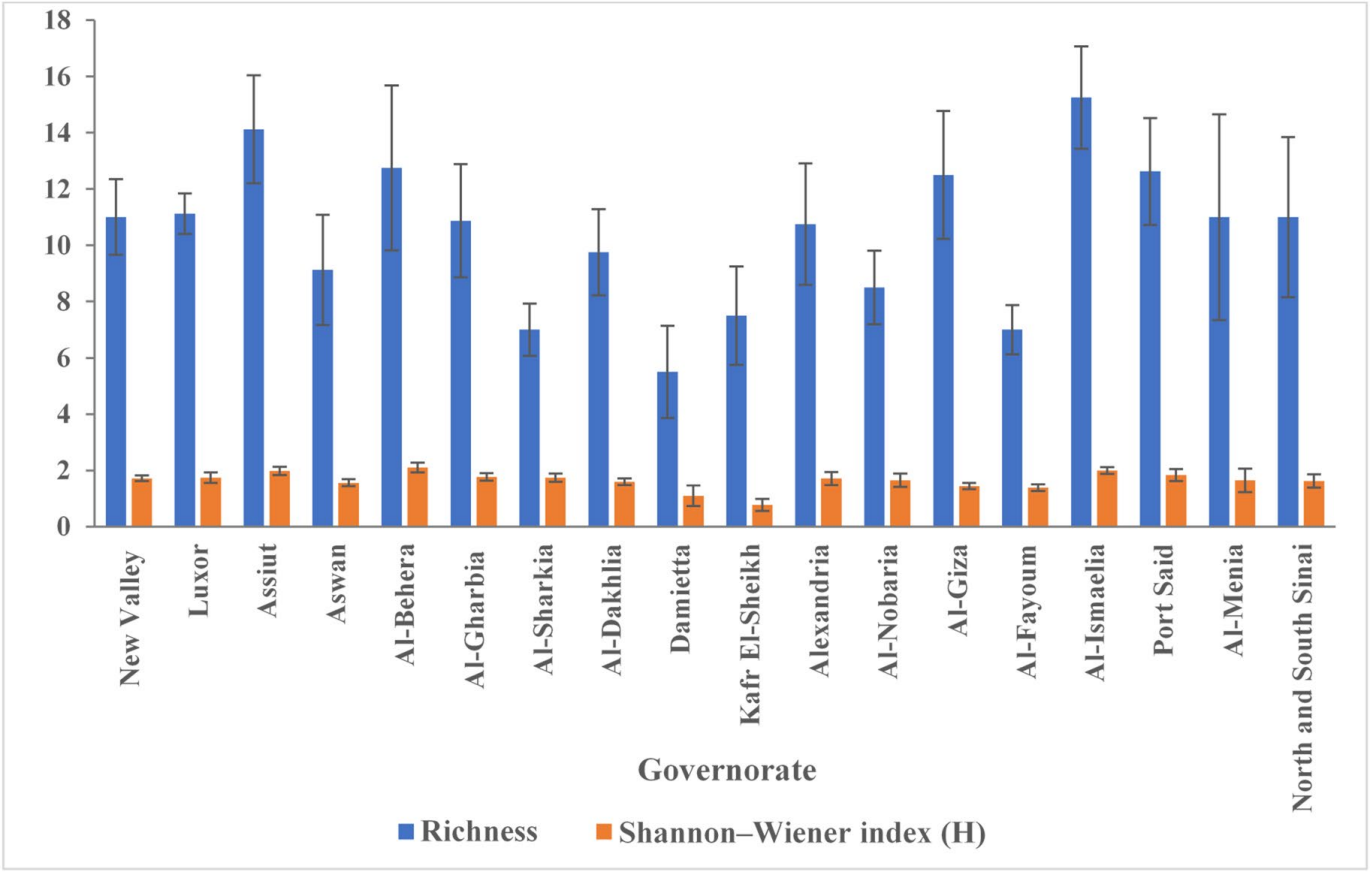
Species richness and Shannon–Wiener diversity index (H) of seed-borne endophytic fungi across Egypt’s 18 maize-growing governorates. The blue bars show the mean number of fungal species (species richness), and the orange bars show the mean Shannon–Wiener index (H), with error bars indicating the standard deviation across sampling sites within each governorate.

At the site level, mean richness per governorate ranged from 5.00 ± 2.07 to 12.75 ± 5.06 species, indicating substantial variability among local assemblages while the mean site-level Shannon diversity indices ranged between 1.09 ± 0.6 and 2.1 ± 0.29, further illustrating regional heterogeneity in species evenness and abundance distributions (Fig. 6). Generally, governorates with higher aggregated richness and diversity also exhibited higher mean site-level indices and lower variability, while less diverse regions showed greater inconsistency across sites. The aggregated results for each governorate, comprising the total number of recovered fungal colonies, the observed species richness and Shannon–Wiener diversity index (H), as well as the mean and standard deviation of these diversity indices across site-level samples, are summarized in Supplementary Table 3 (Table S3). This comprehensive table provides an integrated perspective on both fungal colonization intensity and biodiversity metrics, facilitating inter-regional comparisons.

### Correlations between meteorological factors and the presence of maize endophytic microbiomes

The correlation matrix among meteorological parameters revealed strong interrelationships between key climatic factors (Table 1). A significant negative correlation was observed between temperature and relative humidity (*r* = − 0.81), indicating that increases in temperature were generally associated with decreases in humidity. Temperature was also positively correlated with solar radiation (r = + 0.60), suggesting that warmer conditions coincided with higher levels of sunlight exposure. Other notable correlations included a modest positive association between temperature and wind speed (r = + 0.18) and a negative correlation between relative humidity and solar radiation (*r* = − 0.70). These interdependencies highlight the need to interpret the influence of climatic factors on fungal communities in an integrated manner.

Canonical correspondence analysis (CCA) was conducted to investigate how these climatic variables structured the composition of seed-borne fungi in maize samples across the 18 governorates. The eigenvalues indicated that the first constrained axis explained 36.6% of the total constrained inertia, while the second axis accounted for 27.0%. Together, these axes explained approximately 63.6% of the constrained variation in fungal community structure (Table 2). This proportion underscores the substantial role of climatic conditions in shaping the observed patterns.

**Fig. 7:**
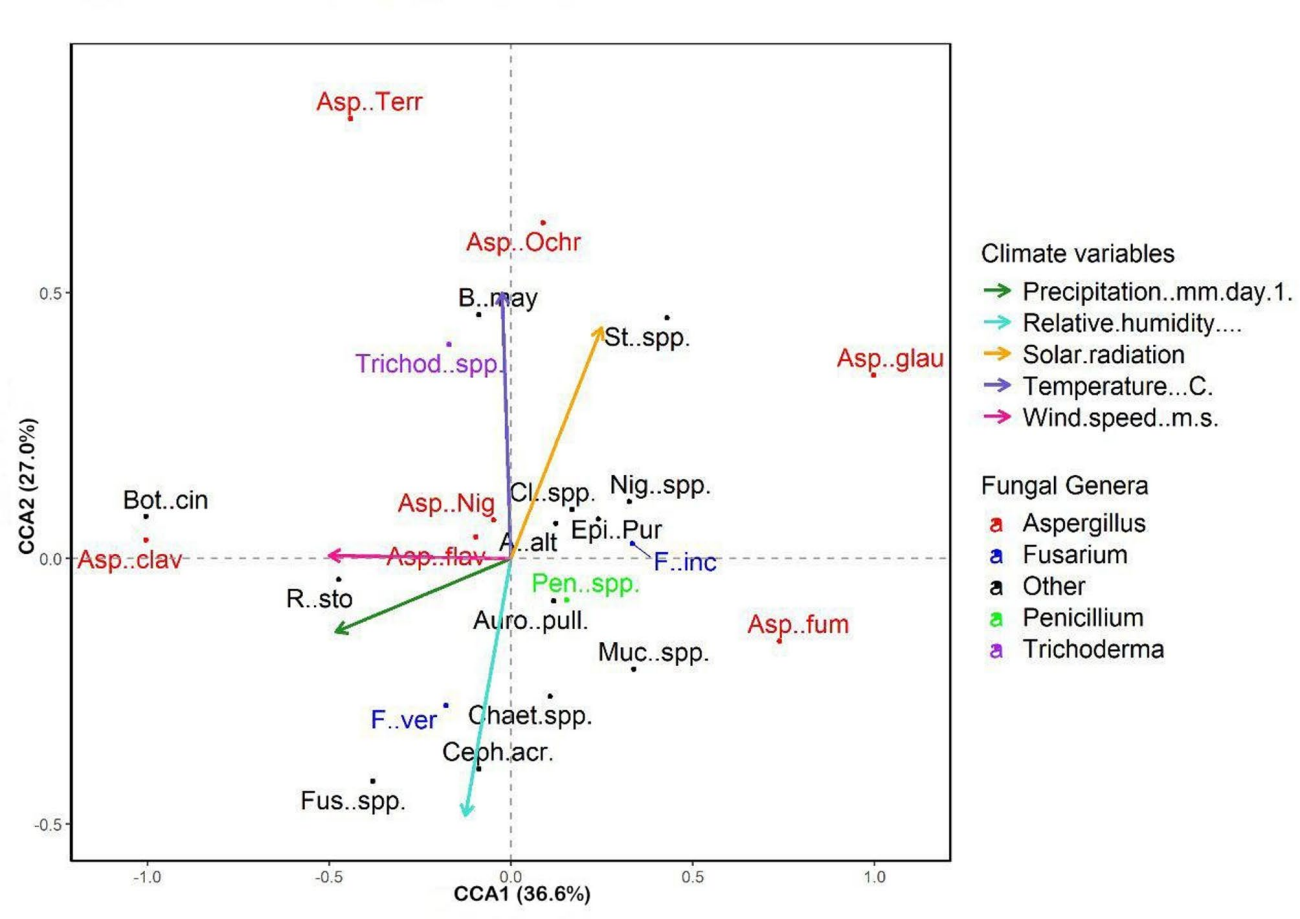
Canonical correspondence analysis (CCA) ordination plot showing the relationship between fungal community composition and selected climate variables across the surveyed governorates. Fungal genera are color-coded: *Aspergillus* (red), *Fusarium* (blue), *Penicillium* (green), *Trichoderma* (purple), and other genera (black). Arrows represent climate variables, each assigned a distinct color: preceptation (forestgreen), relative humidity (turquoise), solar radiation (orange), temperature (slateblue), and wind speed (deeppink). The first two canonical axes (CCA1 and CCA2) explained 36.63% and 27.0% of the constrained variance, respectively. *(A) alt*. (*Alternaria alternata*), *Asp. cla*. (*Aspergillus clavatus*), *Asp. fla.* (*A. flavus*), *Asp. fum*. (*A. fumigatus*), *Asp. gla*. (*A. glaucus*), *Asp. nig*. (*A. niger*), *Asp. och*. (*A. ochraceus*), *Asp. terr.* (*A. terreus*), *Auro. pul.* (*Aureobasidium pullulans*), *(B) may.* (*B. maydis*), *Bot. cin.* (*Botrytis cinerea*), *Ceph. acr.* (*Cephalosporium acremonium*), *Chaet.* spp. (*Chaetomium* spp.), *Cl.* spp. (*Cladosporium* spp.), *Epi. pur.* (*Epicoccum purpurascens*), *F. inc.* (*Fusarium incarnatum*), *Fus. spp.* (*Fusarium* spp.), *F. ver.* (*F. verticillioides*), Muc. spp. (*Mucor* spp.), Nig. spp. (*Nigrospora* spp.), Pen. spp. (*Penicillium* spp.), *R. sto.* (*Rhizopus stolonifer*), *St.* spp. (*Stemphylium* spp.), and *Trichod. spp.* (*Trichoderma* spp.).

The environmental vector fitting (*envfit*) analysis further quantified the relative contribution of each climatic variable to the ordination space **(**Fig. 7**).** Solar radiation exhibited the strongest loading on the second axis (CCA2 = + 0.54), while temperature contributed markedly to both axes (CCA1 = + 0.37; CCA2 = + 0.41). Relative humidity showed a prominent negative association with CCA1 (–0.47), reflecting its contrasting gradient with temperature and solar radiation. Precipitation and wind speed had comparatively smaller vector scores, suggesting more limited effects on the overall fungal assemblages.

The CCA biplot illustrated clear associations between specific fungal taxa and climatic gradients (Fig. 7). *Aspergillus ochraceus*, *Trichoderma* spp., and *A. flavus* clustered along the positive ends of temperature and solar radiation vectors, indicating their preference for warm, high-light environments. Conversely, *Fusarium* spp. and *Stachybotrys* spp. were positioned closer to the relative humidity vector, suggesting a greater reliance on moist conditions.

Species such as *Penicillium spp.* and *Alternaria alternata* showed intermediate positions, reflecting moderate associations with multiple climatic variables. A subset of fungi, including *Epicoccum spp.* and *Botrytis cinerea*, displayed proximity to the wind speed vector, implying potential sensitivity to air movement in dispersal or colonization. In contrast, *A. glaucus* exhibited a lower-than-average requirement for relative humidity but was positively associated with temperature and solar radiation. Overall, while some species demonstrated clear ecological preferences aligned with specific climatic gradients, the biplot did not reveal distinct clustering of sampling sites, indicating that the distribution of fungal taxa was relatively continuous across environmental conditions.

**Table 1:** Pearson moment correlation (r) matrices between the five weather variables recorded from April to August 2021 across Egypt’s 18 governorates that grow maize from which samples of grain were collected (one weather station per governorate).

Variable	Precipitation (mm/day)	Temperature (°C)	Relative humidity (%)	Wind speed (m/s)	Solar radiation (W/m²)
Precipitation (mm/day)	1.00	− 0.77	0.65	− 0.10	− 0.72
Temperature (°C)	− 0.77	1.00	− 0.81	0.12	0.86
Relative humidity (%)	0.65	− 0.81	1.00	− 0.04	− 0.97
Wind speed (m/s)	− 0.10	0.12	− 0.04	1.00	− 0.06
Solar radiation	− 0.72	0.86	− 0.97	− 0.06	1.00

**Table 2:** Canonical correspondence analysis (CCA) results showing eigenvalues, species–environment correlations, and cumulative percentage of variance explained by the first five constrained axes.

Axis	Eigenvalue	Species-environment correlations	Cumulative percentage variance
CCA1	0.06	0.37	36.63
CCA2	0.05	0.27	63.62
CCA3	0.03	0.16	80.01
CCA4	0.02	0.13	93.26
CCA5	0.01	0.07	100.00

Note: Percentages represent the proportion of total constrained inertia explained by each axis. Constrained inertia reflects the variation in fungal community composition that is attributable to the five measured weather variables.

### Morpho-cultural characterization of endophytic *Trichoderma* isolates

**Fig. 8:**
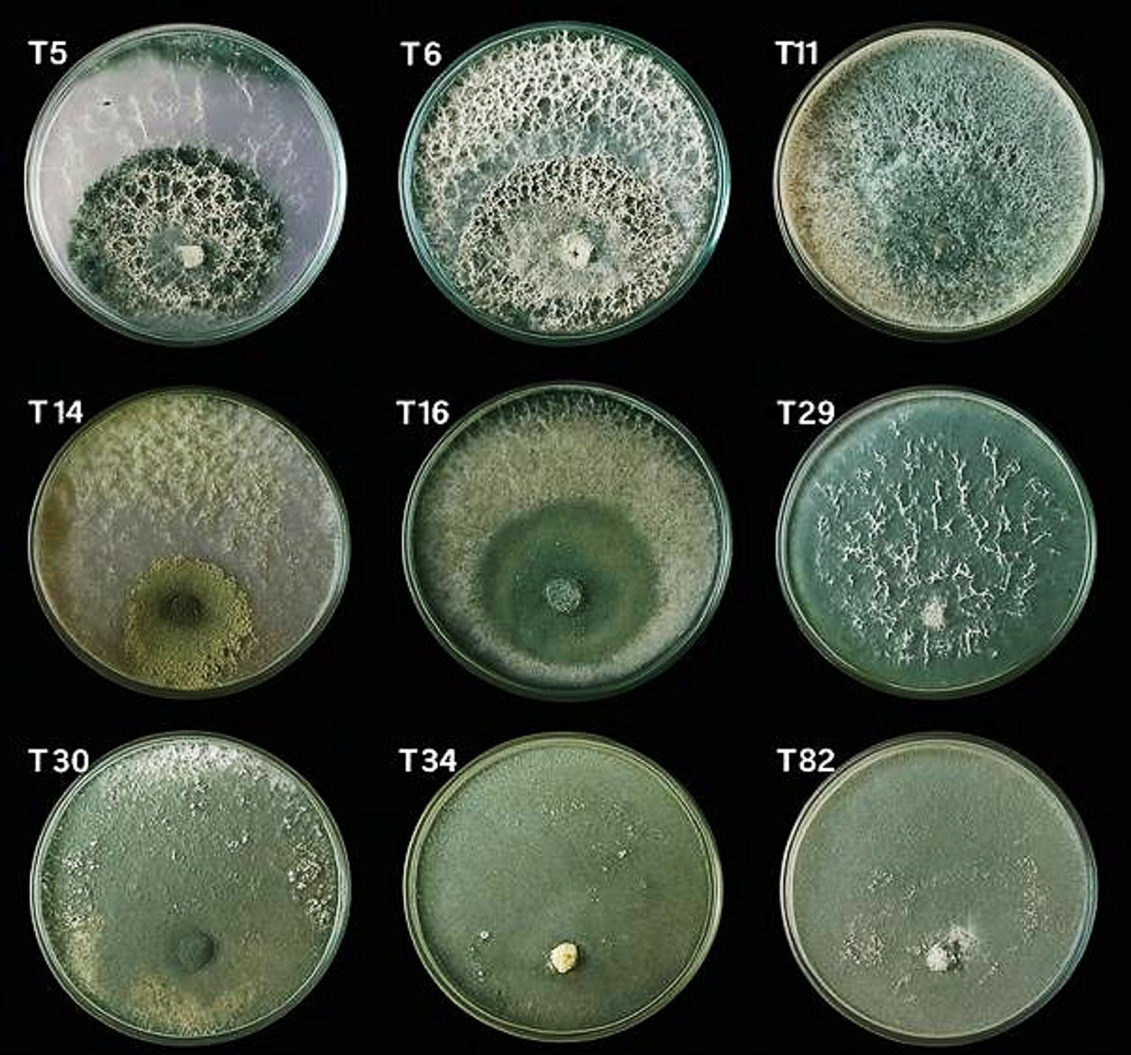
Representative colony morphology of selected *Trichoderma* isolates (T5, T6, T11, T14, T16, T29, T30, T34, and T82) grown on potato dextrose agar (PDA) after 6 days of incubation at 28 °C. All isolates exhibited rapid growth and mycelial development characteristic of the genus.

All fifty *Trichoderma* isolates exhibited rapid growth on PDA, producing colonies typical of the genus, characterized by compact white mycelia that gradually turned green upon conidial formation. Among them, nine representative isolates (T5, T6, T11, T14, T16, T29, T30, T34, and T82) displayed distinct colony morphologies that allowed phenotypic grouping (Fig. 8).

Based on macroscopic and microscopic features, the isolates were classified into three morphological groups (Table 3). Group I (T14, T30, T34, T82) produced cottony colonies with light-green pigmentation and white centers, where conidia were evenly distributed and slightly yellowish near the center. Group II (T5, T6, T11) developed fluffy colonies with dark-green centers showing 1–2 concentric rings of dense conidial growth. Group III (T16, T29) formed velvety colonies with light olive to grey-green pigmentation, characterized by smooth margins and conidia concentrated near the colony centre.

These morpho-cultural traits clearly differentiated the isolates into consistent morphotypes and provided a basis for selecting representative strains for antagonism assays against *F. verticillioides*.

### Dual culture assay

**Fig. 9:**
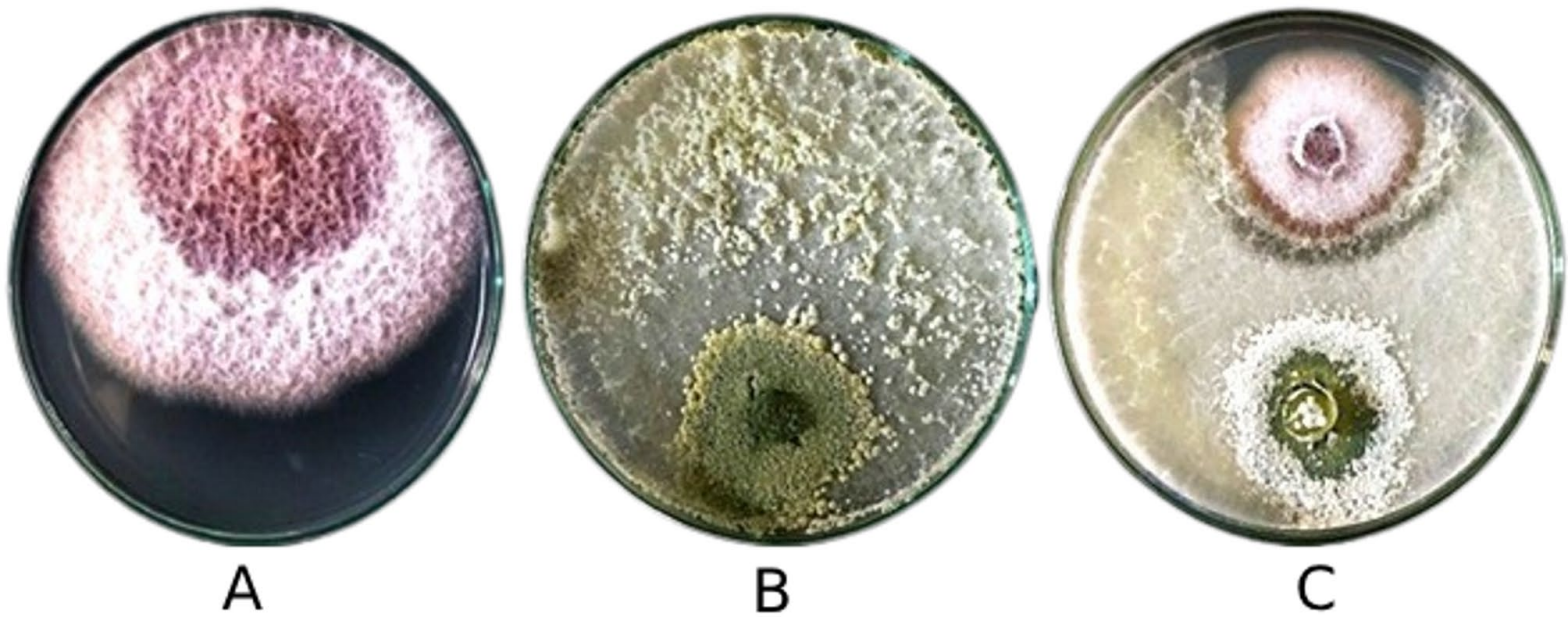
Antagonistic ability of *Trichoderma* endophytic isolate (T14) against *Fusarium verticillioides*, 8th days after inoculation, showing: the growth of *F. verticillioides* mycelium in the control plate (**A**), the growth of *Trichoderma* isolate T14 (**B**), as well as the dual culture of *F. verticillioides* and *Trichoderma* isolate (T14), demonstrating the inhibition and restriction of pathogen growth (**C**).

A dual culture assay was conducted to evaluate the antagonistic potential of 50 *Trichoderma* isolates against *F. verticillioides*. Among these, isolates T14, T30, T34, T16, and T5 demonstrated the most pronounced inhibitory effects, reducing pathogen growth by 74.03%, 73.48%, 71.67%, 71.11%, and 70.16%, respectively, after six days of incubation, most isolates showed reaction type 1 (Table S4). Notably, isolate T14 not only achieved the highest growth reduction but also produced a clear inhibition zone surrounding its colony, indicating strong antifungal activity against *F. verticillioides* (Fig. 9).

**Table 3:** Morphological grouping of endophytic *Trichoderma* isolates based on colony characteristics on PDA after 5 days of incubation at at 25 ± 2 °C, including assessments of colony texture, margins, color, and overall shape.

Group	Texture	Margins	Color	Shape	Representative isolates	Percentage of isolates (%)
I	Cottony	Regular	Light green with white center	Green conidia evenly distributed with yellowish pigmentation near center	T30, T34, T82, T14	44.40%
II	Fluffy	Regular	Dark green with white center	Dense conidial rings concentrated at the center (1–2 concentric rings)	T5, T6, T11	33.30%
III	Velvety	Regular	Light olive to gray-green (non-pigmented)	Smooth colonies with limited green conidia near center or margin	T16, T29	22.20%

### Morphological and microscopic features of *T. longibrachiatum* (T14)

The colony of isolate T14 exhibited rapid growth on PDA, forming abundant white aerial mycelium and prominent green conidial pustules. The colony reverse displayed a light yellowish-green pigmentation, typical of *Trichoderma* spp.

**Fig. 10:**
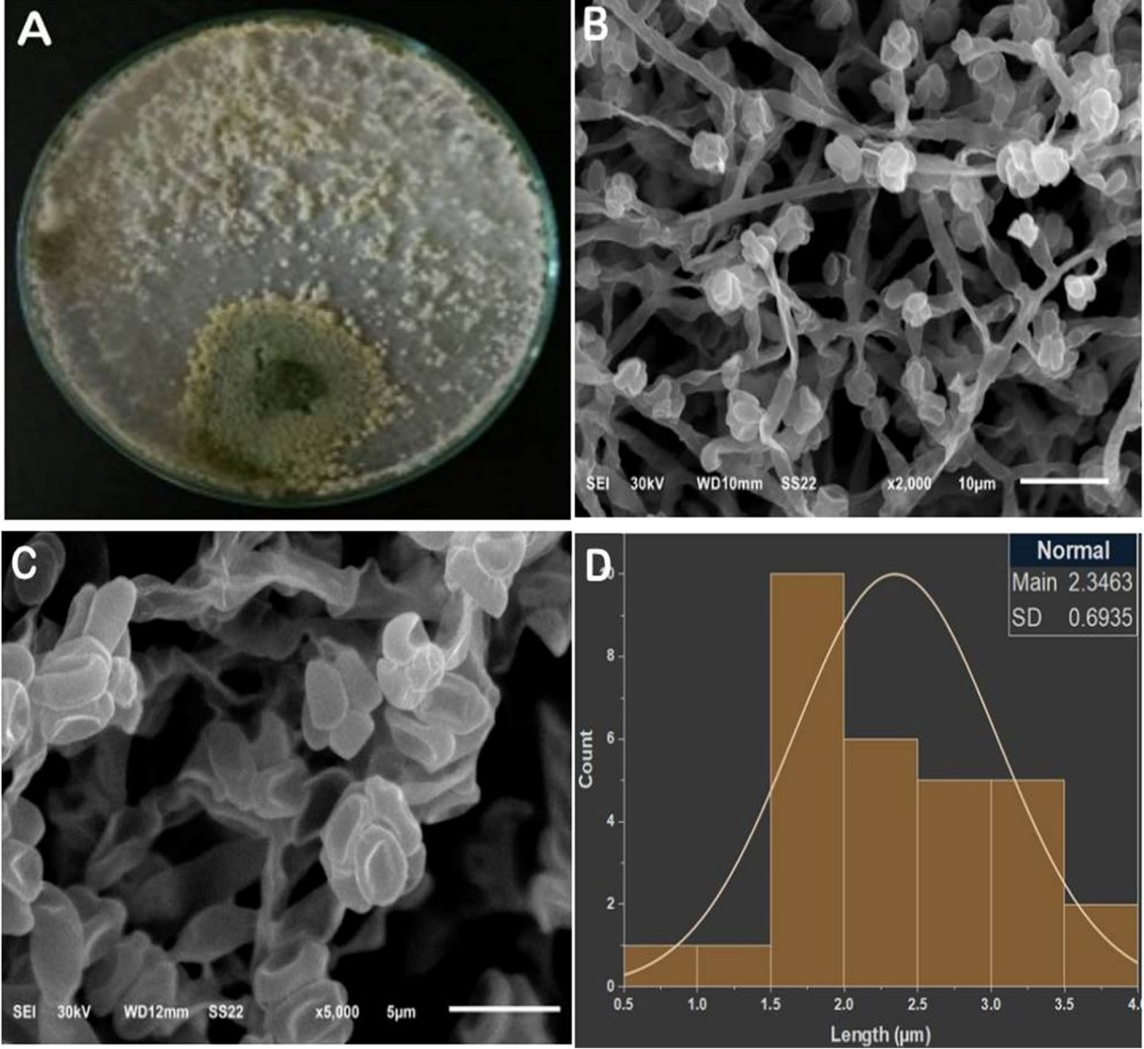
Colony and microscopic features of *T. longibrachiatum* isolate T14. (**A**) Colony on PDA after 7 days of incubation at 25 ± 2 °C showing white aerial mycelium with dense green conidial pustules. (**B**) SEM micrograph (×2000) showing branched conidiophores bearing abundant ellipsoidal conidia. (**C**) SEM micrograph (×5000) showing detailed conidial morphology with smooth wall structure. (**D**) Frequency distribution of conidial lengths (µm) following a normal curve (*n* = 30) generated using Origin Pro (mean = 2.35 μm; SD = 0.6.

Scanning electron microscopy (SEM) revealed branched conidiophores terminating in phialides that bore smooth, ellipsoidal conidia. Quantitative measurements of thirty conidia showed an average length of 2.35 ± 0.69 μm (range: 0.92–3.62 μm), fitting a normal size distribution pattern (Fig. 10). These morphological characteristics are consistent with those of the *T. longibrachiatum* clade. The preliminary identification was verified by the Mycological Centre, Assiut University (AUMC), Egypt.

### Molecular identification

**Fig. 11:**
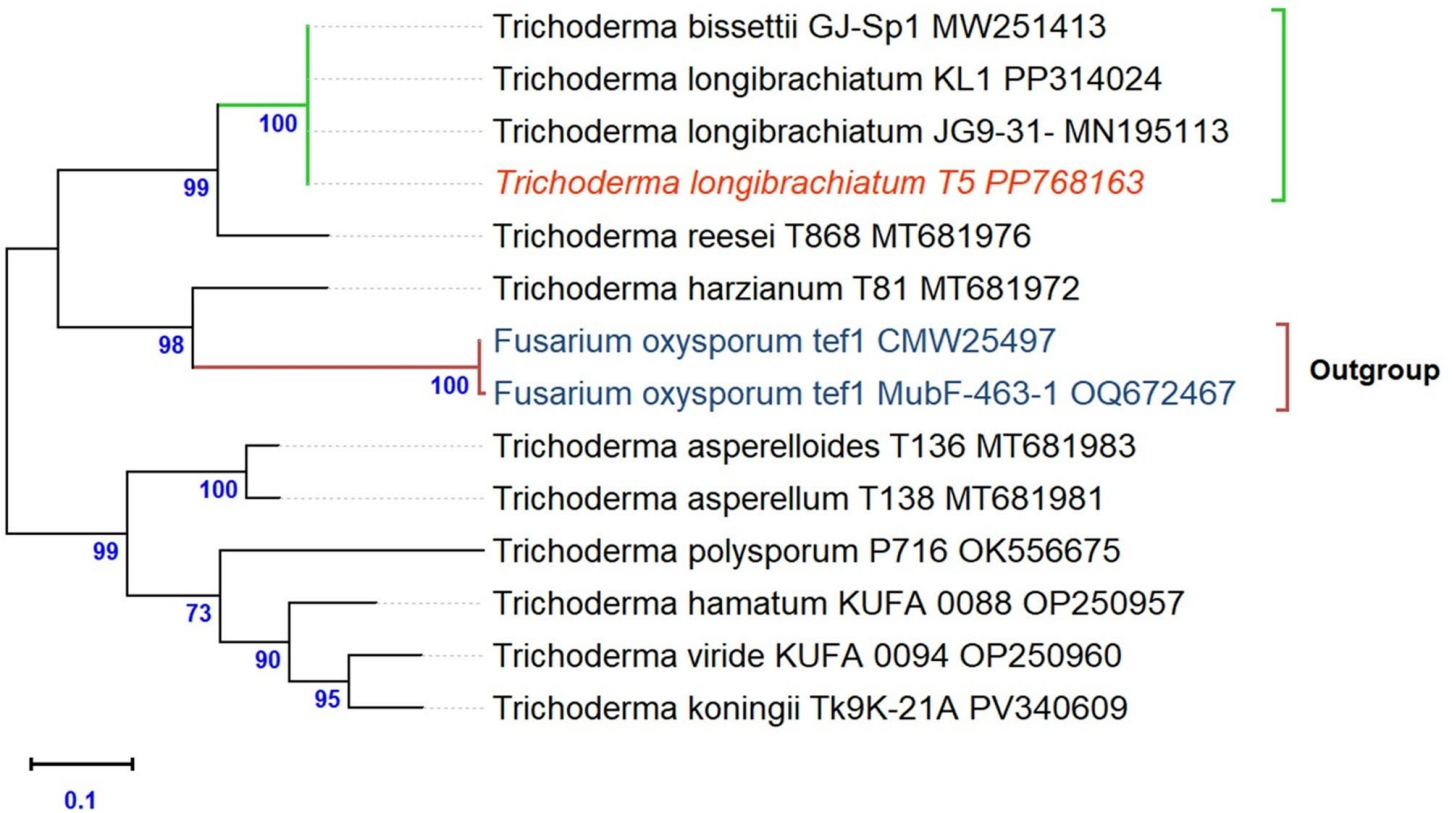
Phylogenetic relationships of *Trichoderma* isolate T14 based on *TEF1-α* sequences. The tree was inferred using the Maximum Likelihood method and Kimura’s 2-parameter model. Bootstrap percentages (1,000 replicates) above 50% are indicated next to the branches. The analysis involved 14 sequences and was performed in MEGA 12. The isolate clustered within the *T. longibrachiatum* clade with high bootstrap support.

BLAST analysis of the partial translation elongation factor 1-alpha (*TEF1-α*) sequence from isolate T14 revealed 100% identity with several *T. longibrachiatum* accessions (e.g., MT881871.1, MN195113.1, KF267252.1) (Table S5). The Maximum Likelihood phylogenetic tree (Fig. 11) clearly placed T14 within a well-supported *T. longibrachiatum* clade, confirming its taxonomic position. High bootstrap values further supported the robustness of this classification. The sequence was deposited in GenBank under accession number PP768163.1.

### Scanning electron microscopy (SEM)

**Fig. 12:**
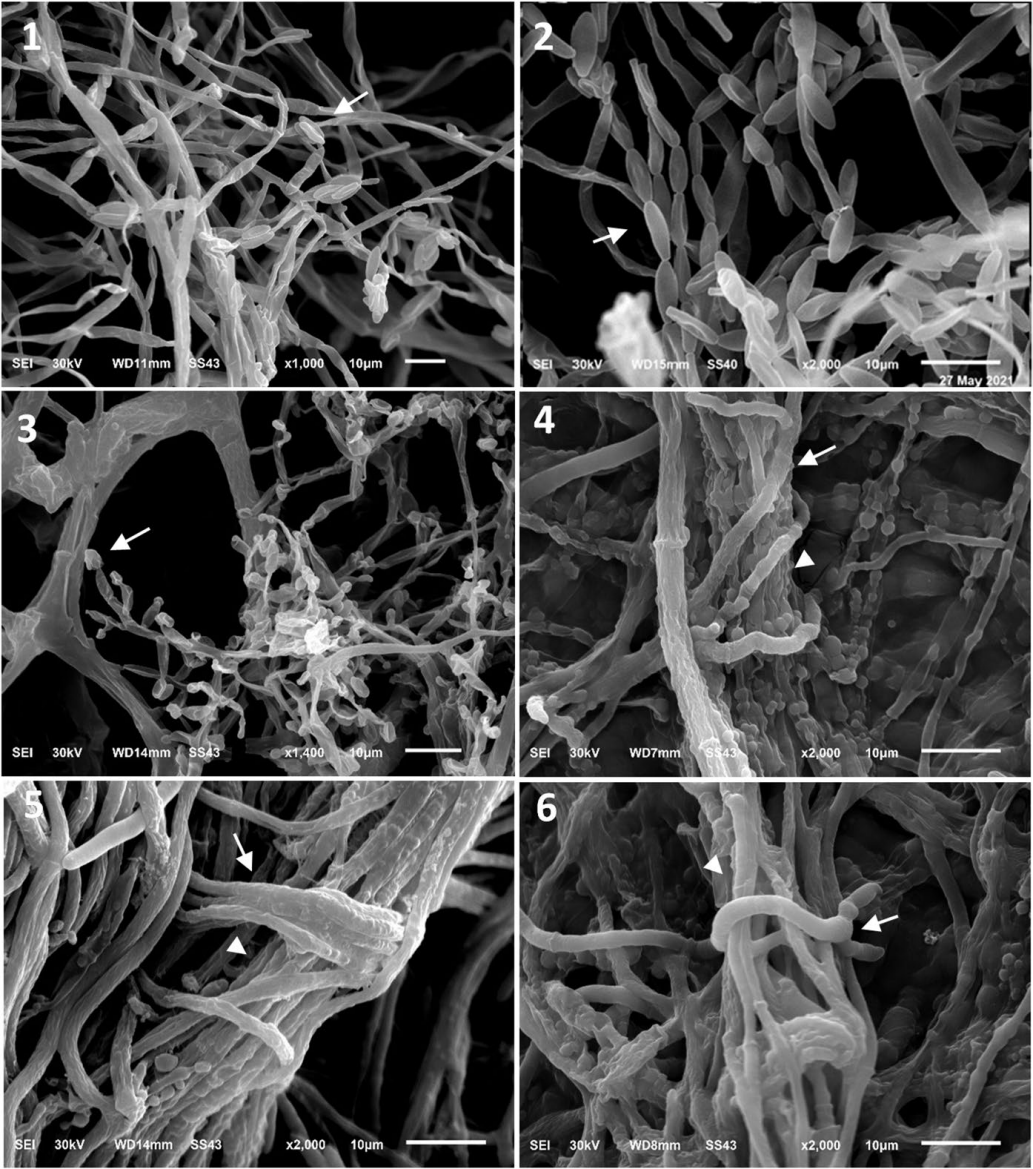
A SEM micrograph demonstrating the parasitic activity of *T. longibrachiatum* (T14) against *F. verticillioides* (*F.V*.). (1) Normal growth of *F.V*. conidiophores and conidia as control. (2) A magnified *F.V*. conidiophores and compacted conidia (arrows). (3) Normal growth of T14 conidiophores and conidia (arrow). (4,5&6) Mycoparasitism process as parallel growth and coiling formation of T14 hyphae (arrow) over the hyphae (arrowhead) of *F.V*. Scale bar = 10 μm.

SEM observations provided direct evidence of the mycoparasitic activity of *T. longibrachiatum* T14 against *F. verticillioides*. The interaction zones revealed multiple specialized structures associated with parasitism, including hook-shaped hyphae, pincer-like projections that appeared to grip the pathogen’s hyphae, and extensive hyphal coilings enveloping the host structures. In addition, abundant conidiogenous cells and densely packed conidia were observed developing along and over the pathogen’s hyphae, suggesting progressive colonization and overgrowth. These features collectively illustrate the complex mechanisms employed by *T. longibrachiatum* during antagonistic interaction (Fig. 12).

## Discussion

This study presents a comprehensive survey of seed-borne endophytic fungi associated with maize grains cultivated across Egypt. A total of 34 fungal species from 23 genera were identified, reflecting a rich and complex mycobiome. Compared to similar studies on legumes such as peanut^42^, common bean and cowpea^43^, or even citrus fruits like Gannan navel orange^44^, maize appears to support a broader endophytic diversity. These comparisons underscore maize’s capacity to harbor diverse fungal endophytes—possibly due to its grain structure, cultivation scale, or environmental adaptability.

Across Egypt’s 18 maize-growing governorates, five fungal taxa *A. flavus*, *A. niger*, *Penicillium* spp., *F. verticillioides*, and *Nigrospora* spp. emerged as a consistent “core” endophyte group, present in nearly every seed sample. A number of additional genera—including *Cladosporium*, *Alternaria*, *Aureobasidium*, *Trichoderma*, *Bipolaris*, *Cephalosporium*, and *Chaetomium*—were also detected, though less uniformly. This pattern of dominance by *Aspergillus*, *Penicillium*, and *Fusarium* species has also been reported in maize grain studies from similar agro-climatic regions. Recent surveys in arid and Mediterranean zones of North Africa and the Middle East, including Saudi Arabia, Tunisia, and Iraq, identified *F. verticillioides*, *A. niger*, and *Penicillium* spp. as core components of maize and cereal seed mycobiota^45–47^. In particular, *F. verticillioides* consistently dominates semi-arid environments due to its tolerance to temperature and moisture fluctuations.

This dominance of *Aspergillus* and *Penicillium* is consistent with previous surveys of maize grains from warm, semi-arid regions, where these fungi thrive due to their resilience to dry conditions and potential for mycotoxin production^48,49^. Similarly, recent studies in Tunisia, Sudan, and Algeria have reported comparable fungal diversity ranges and dominant genera in maize and sorghum seeds, with species richness and Shannon indices reflecting the influence of arid agro-ecologies^50,51^. Additionally, high-throughput sequencing approaches have expanded the known diversity of maize seed mycobiomes, revealing Ascomycota-dominated communities and cryptic taxa alongside culturable genera^52,53^. These findings reinforce the need for integrating culture-based and molecular surveys for a complete ecological understanding.

A recent Tunisian study—conducted under a Mediterranean climate similar to Egypt’s—reported a similar pattern, with *Aspergillus* and *Fusarium* species dominating maize seeds, supporting the existence of a climate-adapted endophyte^54^. Recent Egyptian study reported the biodiversity assessment in maize seeds and revealed that *Ustilago maydis*, *Alternaria alternata*, *Aspergillus flavus*, *A. niger*, *Penicillium* spp., *Cladosporium* spp., and *Fusarium verticillioides* were the most prevalent fungi, each detected in over 90% of samples^55^.

However, the composition of these endophytic communities varied across regions. To explore this spatial variation, we applied the Shannon–Wiener diversity index, which integrates both species richness and evenness, to assess fungal diversity in maize seeds from each governorate^56–59^. The results showed that governorates such as Al-Behera, Luxor, and Al-Menia harbored the most diverse fungal communities, while Damietta exhibited the lowest diversity. These results are fully consistent with a recent study on maize seeds, which reported that Al-Menia Governorate had the most diverse and abundant fungal communities^55^. These differences likely reflect regional variation in microclimate, soil type, and agronomic practices. Similar patterns have been observed in in maize^55^ and other plant species^57–59^, where location, season, and cultivation methods significantly influence the structure of endophytic microbiomes.

The results of the CCA ordination and Pearson correlation analyses revealed distinct associations between specific fungal taxa and climatic variables such as temperature, solar radiation, and relative humidity. However, it is important to emphasize that these relationships are correlative rather than indicative of direct causality. While climatic factors likely play a significant role in shaping fungal community composition, the observed patterns may also be influenced by other environmental and biological factors not accounted for in this study.

Notably, potential confounding factors such as maize cultivar differences, agronomic practices (e.g., seed treatment, irrigation, and fertilization regimes), and soil physicochemical properties could independently or interactively affect the distribution and abundance of seed-borne endophytes. These variables were not controlled for in the present study and may partially explain regional differences in fungal community structure. Future investigations employing multivariate or controlled experimental designs are recommended to disentangle the relative contributions of these factors alongside climatic influences.

CCA revealed that the first two axes explained 36% of the variation, indicating a significant but partial climatic contribution. *Trichoderma* spp. and *A. ochraceus* were associated with higher temperature and solar radiation, while *Fusarium* spp. was linked to more humid conditions. Notably, a strong negative correlation between temperature and relative humidity was observed. These findings are broadly consistent with recent Egyptian research, which reported that *Aspergillus* species are associated with higher temperatures and solar radiation and negatively related to humidity, while *Fusarium* and *Penicillium* showed opposite patterns, and also confirmed the same inverse relationship between temperature and relative humidity using similar CCA approaches^55^. Comparable climate-associated patterns have also been described by Nooh et al.^60^ in stored grains in Egypt, where *Aspergillus* species increased under high temperature and low humidity, while *Fusarium* favoured cooler, humid conditions. Moreover, Brazilian study highlighted that environmental differences play a crucial role in shaping endophytic fungal communities in sorghum, as plants growing in humid regions supported a more diverse and abundant fungal assemblage compared with those from drier environments^61^. Recent studies also emphasize that interactions among temperature, relative humidity, and rainfall are key ecological drivers structuring cereal seed fungal communities in semi-arid systems^45,62,63^. These observations are further supported by previous reports highlighting temperature and humidity as key drivers of fungal distribution^64–66^. Presenting the correlations among meteorological parameters is essential for understanding the interrelationships between these variables. This approach has been widely adopted to illustrate how climatic factors can simultaneously influence fungal growth and dispersal^67,68^. For example, a strong inverse correlation between temperature and relative humidity implies that observed associations with one factor may be partly explained by the other. These findings are further corroborated by studies highlighting that *A. flavus* favours higher temperatures, whereas *F. verticillioides* thrives under more humid conditions^69^. This interpretation aligns closely with recent studies demonstrating that rainfall, temperature, and humidity interactively drive the assembly and diversity of maize seed-borne fungal communities^55,70^. These findings underscore the importance of presenting the intercorrelations among meteorological variables, as they provide essential context for understanding how multiple climatic factors can simultaneously shape fungal composition and prevalence, consistent with the present observation that *Aspergillus* species were linked to higher temperature and *Fusarium* species to more humid conditions. Overall, the results provide strong evidence that maize seeds host a consistent set of endophytic fungi across diverse agroecological zones, with potential significance for agriculture in Egypt’s semi-arid environments.

Although the remaining variation may involve other factors, such as storage practices or maize cultivar, the present results emphasize the importance of climatic conditions, which may contribute to shaping fungal assemblages. This is particularly relevant under projected climate change scenarios^71^.

*Trichoderma* isolates obtained in this study parasitized *F. verticillioides* in dual culture, and isolate T14 was the most aggressive, cutting pathogen growth by 74%. Similar levels of antagonism have been reported for other *Trichoderma* strains^72^, and several species (*T. atroviride*, *T. hamatum*, *T. harzianum*, *T. longibrachiatum* and *T. viride*) have suppressed *F. oxysporum* in vitro and in planta^73–76^.

Antagonistic activity in *T. longibrachiatum* is primarily mediated through the triggering of secondary metabolite pathways, which elicit defense responses against pathogens such as *Fusarium oxysporum*^77^. Furthermore, this species, along with its microbial volatile organic compounds (mVOCs), are widely recognized for their roles in plant growth promotion and biocontrol, contributing to sustainable crop production systems^78^. In the present study, most *Trichoderma* isolates showed strong antagonistic activity against *F. verticillioides*. These findings are consistent with recent reports demonstrating that microencapsulated formulations of *T. longibrachiatum* retain their antagonistic efficacy against *F. oxysporum* under variable environmental conditions^79^.

BLASTN analysis of this study placed T14 within the *T. longibrachiatum* clade with 100% identity to reference strains, indicating very low intraspecific divergence^80,81^.

In this context, the *TEF1-α* gene was selected as the primary molecular marker due its superior taxonomic resolution compared with *ITS* region^82^. We acknowledge that formal taxonomic frameworks for description of new species typically require a multi-locus approach, incorporating markers such as *ITS*,* TEF1-α*,* and rpb2*. However, within the *Longibrachiatum* clade, single-locus phylogenies have been shown to be highly congruent and reliable^83^. Accordingly, and given the limitations in sample resources in the present study, we adopted an approach consistent with recent applied investigations^84^, in which species identification based on high-quality *TEF1-α* sequencing, supported by morphological characterization, was considered scientifically robust and adequate for achieving the biocontrol objectives of this study.

Scanning-electron microscopy corroborated its mycoparasitic strategy: hyphal coils and wrapping structures enveloped *F. verticillioides* hyphae—features consistent with the established mechanisms of *Trichoderma* species, which define their ability to coil around pathogens and produce appressoria-like structures^85^. The coiling around the pathogens, conidial attachment, and other mechanisms of *T. longibrachiatum* revealed the mycoparasitic effect on *Fusarium oxysporum*,* Fusarium solani* and *Fusarium pseudograminearum* growth^86,87^. Based on the present findings, *T. longibrachiatum*, a seed-borne endophyte isolated from maize across diverse agroecological zones in Egypt, shows strong in vitro potential as a biocontrol agent against *F. verticillioides*.

## Conclusion

This study provides a comprehensive assessment of seed-borne fungal endophytes associated with Egyptian maize across diverse agroecological zones. Thirty-four species were identified, with *F. verticillioides*, *A. flavus*, and *Penicillium* spp. forming a consistent core community across most regions. Canonical ordination analysis revealed that temperature, solar radiation, and relative humidity were the main climatic factors shaping endophytic community structure, underscoring the ecological responsiveness of maize-associated mycobiota. Among the isolates, *T. longibrachiatum* exhibited the strongest antagonistic activity, reducing *F. verticillioides* growth by nearly 74%, highlighting its potential as a promising native biocontrol candidate for maize cultivation in semi-arid regions.

### Limitations

Despite these valuable insights, the study has certain limitations. Sampling was conducted during a single growing season and from a limited number of sites, which may not fully capture temporal and spatial variability in fungal communities. The identification was primarily based on culture-dependent methods, potentially excluding unculturable or cryptic taxa, and molecular confirmation was limited to *T. longibrachiatum*. Moreover, non-climatic variables such as soil properties, maize cultivar differences, and agronomic practices were not directly evaluated, though they likely influence the observed distribution patterns. Finally, the relationships identified between community composition and climatic factors are correlative rather than causal and should therefore be interpreted with caution.

### Future directions

Future research should integrate culture-independent sequencing approaches (e.g., *ITS*, *TEF1-α*, and metagenomics) with multi-seasonal and multi-location sampling to achieve higher taxonomic resolution and ecological accuracy. Field validation of *T. longibrachiatum* T14, including formulation optimization and seed-coating trials, will be essential to validate its efficacy and develop endophyte-based biocontrol strategies tailored to Egyptian agroclimatic conditions.

## Methods

### Area of study

**Fig. 13:**
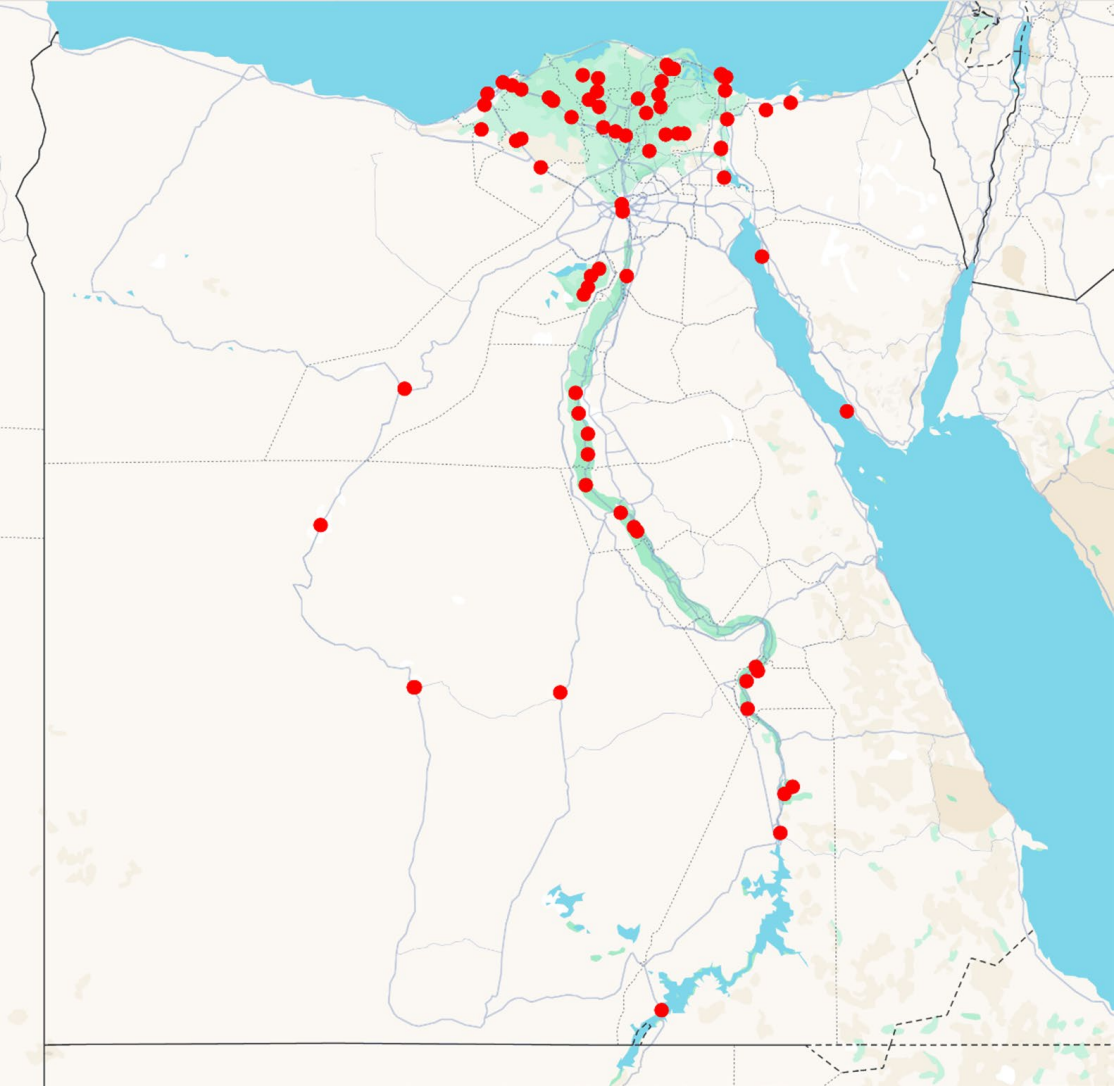
Map showing the locations of maize grain sampling sites across 18 maize-producing governorates in Egypt. Each red dot represents one of the sampling sites, with four villages sampled per governorate.

In 2021, a survey was carried out in Egypt’s governorates that grow maize from April to August and the geographical coordinates of all sampling locations were recorded using a global positioning system (GPS), and a map of sampling 18 governorate sites (4 villages for each) was created ArcGIS software (version 10.1; Environmental Systems Research Institute (ESRI), 2012; Redlands, CA, USA; https://www.esri.com/en-us/arcgis/products/arcgis-desktop/overview) (Fig. 13). The area of study spans latitudes between 22°33′ N and 33°975′ N, and longitudes between 27°96′ E and 33°83′ E., encompassing diverse agroecological zones ranging from arid desert regions to Mediterranean coastal areas.

### Weather-related conditions

Egypt is predominantly influenced by a desert climate, ranging from semi-arid to dry. Egypt’s climate can be classified into four distinct zones: the arid desert in the south, the semi-desertic in the middle, the Mediterranean climate along the northern shore, and the red sea climate zone on the eastern coast. Upper Egypt experiences a tropical climate. The northern coastal area experiences a constant north-westerly wind from the Mediterranean Sea, which leads to moderate temperatures all year round. In contrast, the central and southern regions experience different conditions, particularly with higher nighttime temperatures during the summer. A powerful, hot, dusty, and dry wind known as *Khamasīn*, that originates from the south or southwest affects the country every year from March to May, causing temporary spikes in temperatures and a sharp drop in humidity below 5%. Along the Mediterranean coast, Egypt receives an average annual precipitation of 20 to 200 mm, while the central and southern regions, including Cairo, experience an average of approximately 0 mm. The country enjoys substantial annual sunshine, ranging from 3300 h in the north to 4000 h in the interior. During the maize growing season from April to August 2021, the average temperature ranged from 25.5 to 32.9 ^◦^C, the relative humidity was between 18.8 and 75.2%, the solar radiation levels ranged between 0.67 and 0.73 W/m², the wind speed varied between 4.25 and 5.18 m s^− 1^, and the rainfall measured between 0 and 0.16 mm day^− 1^^88^.

### Method of sampling

Samples of maize grains were collected from eight sites within each governorate. At each site, at least fifty corncobs from local white and yellow maize hybrids were randomly selected, and grains were manually separated. Samples were transported in sterile bags placed in a cold box to maintain sample integrity. Upon arrival at the laboratory, grains were spread on clean trays and left to dry at a room temperature (25 ± 2 °C) for seven days prior to processing.

### Survey and isolation of endophytic fungi from maize grains

For isolation of endophytic fungi, healthy maize grains were surface sterilized by soaking in 70% ethanol for 2 min, followed by 2% sodium hypochlorite (NaOCl) for 5 min, and then 70% ethanol for 0.5 min^89^. The grains were washed several times with sterile water three times each for 1 min, allowed to air-dry under aseptic conditions and then the efficacy of sterilization was confirmed by imprinting the final rinse on potato dextrose agar (PDA) medium. A total of 200 grains were plated per site (10 grains per Petri dish) on PDA and incubated at 22 ± 2 °C for 7 days^90^. All endophytic fungi obtained were recorded and isolated in pure cultures using either the single-spore or hyphal-tip techniques. Identification of the isolates was based on morphological and cultural characteristics by experienced mycologists based on standard taxonomic keys as well as colony characteristics and microscopic features^91–94^. Molecular sequencing was not performed due to the large-scale nature and scope of the diversity assessment.

The following formulae were used to determine the colonization and frequency of each endophyte species:

1 $${\text{Colonization }}\left( \% \right)=\left( {\frac{{{\text{Number of colonies of the species}}}}{{{\text{Total EquationNumber of seed tested}}}}} \right) \times {\mathrm{1}}00$$

Frequency was calculated at two levels: (i) per-governorate frequency, representing the proportion of sites within each governorate where each species occurred, and (ii) national frequency, representing the proportion of all sampling sites across all governorates where each species was detected. Frequency (%) of occurrence was defined as the proportion of sampling sites in which the species was detected:

2 $${\text{Frequency }}\left( \% \right)=\left( {\frac{{{\text{Number of sites in which the species occurred}}}}{{{\text{Total EquationNumber of sites}}}}} \right) \times {\mathrm{1}}00$$

### Measures of fungal diversity

Diversity indices, including species richness and Shannon–Wiener diversity (H), were initially calculated at the site level, based on the fungal community recovered from 200 grains per site. For each governorate, the mean and standard deviation (Mean ± SD) of these eight site-level values were computed to capture within-governorate variability. Additionally, a national-level diversity index was derived by aggregating data from all 144 site-level samples across the 18 governorates.

Fungal diversity measures were taken to assess the variety of endophytic fungal species found in the maize samples gathered throughout Egypt. Using endophytes seed health testing data gathered throughout Egypt, frequency (calculated using Eq. (1) and relative abundance (evenness, %) were evaluated on a national scale. The following equation was used to estimate relative abundance:

3 $${\text{Relative abundance }}\left( \% \right)=\left( {\frac{{{\text{Number of species infected with a given fungal species}}}}{{{\mathrm{Total}}\,\,{\mathrm{EquationNumber}}\,\,{\mathrm{of}}\,\,{\text{species infected with all IDentified fungal species}}}}} \right) \times {\mathrm{1}}00$$

The richness of fungal species and the Shannon–Wiener diversity index (H) was calculated for each of the 18 maize-growing governates. Species richness was determined as the total number of the fungal species identified within a governate: Species richness = total number of distinct fungus species in a maize-growing governate.

The Shannon–Wiener diversity index (H), incorporating species richness and relative abundance, was calculated using the formula:

4 $${\mathrm{Shannon}}-{\text{Wiener diversity index }}\left( {\mathrm{H}} \right)\,=\, - {\,_{{\mathrm{i}}={\mathrm{1}}}}{\sum ^{\mathrm{S}}}{\mathrm{Pi}} \times {\mathrm{Ln}}\left( {{\mathrm{Pi}}} \right)$$

where Pi = ni/N (ni is the number of grains with the species identified I and N is the total number of grains with all fungal species identified), representing relative abundance in fractional form. Ln refers to the natural logarithm.

Richness and Shannon diversity indices were calculated at three hierarchical scales: per site (individual sampling location), per governorate (aggregated across the eight sites within each governorate), and nationally (aggregated across all samples). Within each governorate, the mean and standard deviation (Mean ± SD) of both richness and Shannon diversity were computed from the eight site-level values to quantify within-region variability in fungal community structure.

Distribution maps were developed for the occurrence of key endophytic seed-borne fungi of maize using the software R (2020) and the packages “raster”^95^, “sp”^96^, and “ggplot2”^97^.

### Antagonistic effect of *Trichoderma* spp

From the collected endophytic fungal isolates, fifty *Trichoderma* isolates were selected based on their high colonization frequency and distinct morphological features to maximize diversity among the recovered strains. Preliminary genus-level identification was conducted in our laboratory under the supervision of mycological experts, following the criteria described by^98^.

The antagonistic activity of the isolates against *F. verticillioides* was evaluated using the dual culture technique^99^. The pathogen isolate, obtained from the Agricultural Research Center, Giza, Egypt, was identified as *F. verticillioides* (GenBank: OM835927). For each assay, 5-mm mycelial plugs from seven-day-old cultures of both *Trichoderma* and *F. verticillioides* were placed on opposite sides of PDA plates at a 5-cm distance. Plates were incubated at 25 ± 2 °C for six days under a 12-hour photoperiod^100^.

Each *Trichoderma* isolate was tested in quadruplicate, with control plates containing only the pathogen. Representative isolates were maintained under sterile conditions for subsequent studies.

After six days of incubation, pathogen growth inhibition was calculated relative to the control using the following formula^101^:

5 $${\text{Growth inhibition }}\left( \% \right)=\left[ {\left( {{{\mathrm{R}}_{\mathrm{1}}} - {{\mathrm{R}}_{\mathrm{2}}}} \right)/{{\mathrm{R}}_{\mathrm{1}}}} \right] \times {\mathrm{1}}00$$

where R_1_ is the pathogen colony radius in the control plate and R_2_ is the pathogen colony radius toward *Trichoderma* in the dual culture plate.

Between the eighth and tenth day of incubation, fungal interactions were scored on a 1–5 scale, representing levels of overgrowth and inhibition, following Bell et al.^102^. Colony radius was used as a practical and widely adopted metric to estimate fungal growth^103^. Data are presented as mean ± standard error (SE), and statistical significance was determined using ANOVA as described in the Statistical Analysis section.

### Identification and morphological characterization of *Trichoderma* T14 isolate

The most effective *Trichoderma* isolate (T14), selected for its strong antagonistic activity against *F. verticillioides*, was cultured on PDA (DIFCO, Tucker, GA, USA) and incubated at 25 ± 2 °C for seven days. Colony characteristics, including color, texture, and growth pattern, were recorded. Preliminary morphological identification was performed according to the diagnostic criteria described by Rifai^104^ and Bissett^105^.

For taxonomic verification, the isolate was submitted to the Mycological Centre, Assiut University (AUMC), Egypt, where expert examination confirmed its identity as *T. longibrachiatum* Rifai (AUMC No. 16199). Quantitative analysis of conidial size was conducted using ImageJ software (version 1.54 g; NIH, USA; https://imagej.net/ij/). Thirty randomly selected conidia were measured from high-resolution SEM micrographs captured at ×2000 and ×5000 magnifications.

Descriptive statistical parameters (mean, standard deviation, range) were calculated using OriginPro 2025b (version 10.2.5.212; OriginLab Corp., USA; https://www.originlab.com/) to evaluate size variation and confirm morphological consistency.

### Molecular identification and phylogenetic analysis of efficient antagonistic *Trichoderma* [T14]

To confirm the identity of the T14 isolate, molecular identification was conducted by targeting the translation elongation factor 1-alpha (*TEF1-α*) gene. This marker was specifically selected for species-level diagnosis due to its high conservation and superior phylogenetic resolution in *Trichoderma* compared with the *ITS* region, in accordance with authoritative taxonomic guidelines^82^ and recent applied protocols^84^.

Fungal isolate T14 was cultured on PDA at 25 °C for 7 days, from which total genomic DNA was extracted following the manufacturer’s protocol for the Genomic DNA Isolation (Plant) Kit (GeneDireX, cat no. NA025-0100). The TEF region of the fungus was amplified using PCR with specific primers for the TEF1-alpha gene (EF1-728 F: CATCGAGAAGTTCGAGAAGG, EF1-986R: TACTTGAAGGAACCCTTACC) on a Labnet MultiGene™ Gradient PCR thermal cycler. The PCR reaction was set up in a total volume of 25 µl, containing two microliters of DNA template, one microliter of each primer, 12.5 µl of PCR master mix, and two microliters of MgCl_2_ (25 mM). The PCR amplification conditions included an initial denaturation cycle at 95 °C for 5 min, followed by 35 cycles of denaturation at 95 °C for 30 s, annealing at 56 °C for 45 s, and extension at 72 °C for 40 s. The final extension was performed at 72 °C for 5 min. The amplified products were analyzed using electrophoresis on a 1.2% agarose gel. The resulting amplicons were purified and sequenced by Macrogen Inc. (Seoul, South Korea) using the Sanger method. Sequence identity was determined using the BLAST tool (https://blast.ncbi.nlm.nih.gov) to compare with GenBank entries.

Phylogenetic relationships were inferred using the Maximum Likelihood method implemented in MEGA version 12^106^, based on the Kimura two-parameter model of nucleotide substitution^107^. Sequence alignment was conducted using the MUSCLE algorithm, and branch support was assessed with 1,000 bootstrap replicates^108^.

It is acknowledged that molecular identification was limited to isolate T14 due to resource constraints, while the remaining isolates were identified morphologically. This is recognized as a limitation of the present study and will be addressed in future work.

The representative *Trichoderma* isolates used in this study are securely maintained in the Seed Pathology and Tissue Culture Laboratory, Faculty of Agriculture, Mansoura University, Egypt, where they are available upon reasonable request. We are planning to deposit these cultures in an accredited national or international culture collection facility in the near future to enhance reproducibility and access.

### Ultrastructural analysis of the antagonistic interaction between *Trichoderma* [T14] and *F. verticillioides* using scanning electron microscopy

To visualize the antifungal interactions at the microstructural level, mycelial samples were collected from three zones: (1) the inhibition zone in the dual culture assay of *Trichoderma* T14 against *F. verticillioides*, (2) the actively growing edge of *Trichoderma* T14 (control), and (3) the actively growing edge of *F. verticillioides* (control). All samples were collected simultaneously from the most recent growth areas to ensure consistency in developmental stage. Sample preparation for SEM followed the method described in^109^. Briefly, samples were initially fixed in a solution of 2.5% glutaraldehyde and 2% paraformaldehyde in 0.1 M sodium phosphate buffer (pH 7.4) at 4 °C for 12 h. After three rinses in phosphate buffer with 0.1 M sucrose (15 min total), post-fixation was carried out using 2% osmium tetroxide for 90 min. Samples were then rinsed and dehydrated through a graded ethanol series (30–100%). After critical point drying, specimens were coated with a gold-palladium film and observed using a Jeol JSM-6510 L.V scanning electron microscope.

### Statistical analyses

All statistical analyses were performed using CoStat (version 6.45; CoHort Software, Monterey, CA, USA; https://www.cohortsoftware.com/costat.html). Prior to conducting one-way analysis of variance (ANOVA), data were tested for normality using the Shapiro–Wilk test and for homogeneity of variances using Levene’s test. Only datasets meeting these assumptions were subjected to ANOVA. When significant differences were detected (*p* ≤ 0.05), means were compared using Tukey’s Honestly Significant Difference (HSD) test^110^ for multiple comparisons to identify statistically distinct groups. The significance level was set at *p* ≤ 0.05 for all tests.

The incidence and frequency of the recovered maize seed-borne mycoflora were assessed using Excel software to create a heatmap. Additionally, Canonical correspondence analysis (CCA) was performed using R software (version 4.5.1; https://www.r-project.org/) with the “vegan” package (version 2.7-1; https://cran.r-project.org/web/packages/vegan/index.html) to determine the effects of meteorological variables on the occurrences of fungi transmitted by maize seeds^111^. The analyzed meteorological variables included monthly average precipitation (mm day⁻¹), daily mean air temperature (°C), relative humidity (%), wind speed (m s⁻¹), and solar radiation (W m⁻²).

## Supplementary Information

Below is the link to the electronic supplementary material.

Supplementary Material 1

## Data Availability

All data generated or analyzed during this study are included within the published article and its supplementary materials. The sequencing data have been deposited in the NCBI database, with the ITS sequence under GenBank accession number PP768163.1. The fungal material used in this study was formally identified by Dr. Khalid M. Ghoneem Plant Pathologist and Dr. Yasser M. Shabana Plant Pathologist. A voucher specimen was not required for deposition in a publicly accessible herbarium for this study.

## Abbreviations

SEM: Scanning electron microscopy
GPS: Global positioning system
CCA: Canonical correspondence analysis
TEF1-α: Partial translation elongation factor 1-alpha
