# Supplementary material for: Diversity, climatic correlations, and biocontrol prospects of seed-borne fungal endophytes in Egyptian maize
Source: Sci Rep. 2026 Mar 26;16:10371. doi: 10.1038/s41598-026-41567-3 (PMC13031278; doi:10.1038/s41598-026-41567-3)
Supplement: Supplementary file 1 — Supplementary Material 1 [file 41598_2026_41567_MOESM1_ESM.docx]

**Supplementary tables**

**Diversity, Climatic Correlations, and Biocontrol Prospects of Seed-Borne Fungal Endophytes in Egyptian Maize**

Khadiga A. Hasan^a^, Hoda M. Soliman^a^, Khalid M. Ghoneem^b*^ and Yasser M. Shabana^c*^

^a^Botany Department, Faculty of Science, Mansoura University, Mansoura 35516, Egypt; khadiga.ahmed.php@gmail.com (K.A.H.); [dr.hodasoliman_1964@yahoo.com](mailto:dr.hodasoliman_1964@yahoo.com) (H.M.S.)

^b^Seed Pathology Research Department, Plant Pathology Research Institute, Agricultural Research Center, Giza 12619, Egypt; [khalidghoneem@arc.sci.eg](mailto:khalidghoneem@arc.sci.eg); https://orcid.org/0000-0003-4629-6399 (K.M.G.)

^c^Department of Plant Pathology, Faculty of Agriculture, Mansoura University, Mansoura 35516, Egypt; [yassershabana2@yahoo.com](mailto:yassershabana2@yahoo.com) (Y.M.S.)

Correspondence, [khalidghoneem@arc.sci.eg](mailto:khalidghoneem@arc.sci.eg) (K.M.G.); [yassershabana2@yahoo.com](mailto:yassershabana2@yahoo.com) (Y.M.S.)

**Table S1. Morphological and cultural characteristics of endophytic fungi isolated from maize seeds.**

| **Fungus** | **Colony** | **Conidiophore** | **Conidia** |
| --- | --- | --- | --- |
| ***Absidia* spp*.*** | White, rapidly growing colonies reaching over 3.1 cm in height after 4 days at 25 °C on PDA. | Sporangiophores 3.4–5.0 µm wide, branched in whorls of short branchlets, each terminating in a single sporangium. | Sporangia are pyriform (34.4 × 26.3 µm), apophysate; sporangiospores are spherical (2.2–3.4 µm); columellae hemispherical with slight apical projection. |
| ***Alternaria alternata*** | Golden brown colonies, 6 cm diameter after 7 days at 25 °C on MEA. | Simple, straight, septate, up to 50 µm long and 3–6 µm wide. | Ovoid, brown conidia with short apical beaks and 1–2 longitudinal septa, 18–63 × 7–18 µm. |
| ***Arthrobotrys* spp.** | Whitish cottony colonies with radial mycelial growth after 14 days at 25 °C. | Conidiophores 32–100 µm long, single or in clusters, emerging from hyphal bundles. | Obovoid to ellipsoidal, 1-septate conidia grouped in clusters of 4–5 at apices, 8.9–17.8 × 3.1–5.8 µm. |
| ***Aspergillus clavatus*** | Bluish-green colonies, 3.5 cm after 10 days at 25 °C on CzA, reverse initially colourless becoming brown. | Clavate conidial heads (320–400 × 160–200 µm) that split into compact divergent columns; conidiophores 1.7–3.0 mm × 22–29 µm, smooth-walled. | Conidia elliptical, smooth, thick-walled (3.2–4.3 × 2.7–3.5 µm). Cleistothecia form after 8 weeks, 350–690 µm, enclosing lenticular ascospores (6.4–6.9 µm). |
| ***Aspergillus flavus*** | Yellow-green colonies, 5 cm on CzA and 7 cm on MEA after 10 days at 25 °C. | Conidiophores hyaline, 0.6 mm, rough-walled, bearing radiating conidial heads with phialides and metulae. | Conidia are globose, finely roughened, 3–4 µm in diameter. |
| ***Aspergillus fumigatus*** | Blue-green, suede-textured colonies, 4 cm after 7 days at 25 °C on CzA. | Columnar conidial heads (400 × 50 µm), uniseriate; conidiophores short, smooth; vesicles conical with a single phialide row. | Conidia are globose to subglobose, echinulate, 2.6–2.9 µm, forming long chains basipetally. |
| ***Aspergillus glaucus*** | Colonies grayish to turquoise-green with yellow centers; reverse yellow to pale brown on PDA. | Septate hyphae; smooth conidiophores (260–340 µm), with uniseriate globose vesicles (19–25 µm) bearing phialides. | Conidia rough, 3.5–6.5 µm; yellow cleistothecia (80–120 µm) with red hyphae; asci 6.2–6.9 × 3.8–4.8 µm. |
| ***Aspergillus niger*** | Black, powdery colonies, 3–6 cm after 10 days at 25 °C on CzA and MEA. | Conidiophores arise from brownish foot cells, 1.5–3.0 mm tall. | Conidia globose, irregularly roughened, 4–5 µm, forming large radiating heads. |
| ***Aspergillus ochraceus*** | Light buff colonies, 3 cm on CzA and 5 cm on MEA after 12 days at 25 °C. | Conidiophores yellow to light brown, 1.5 mm long, coarsely rough, ending in globose vesicles with metulae. | Conidia are globose, finely roughened, 2.5–3 µm in diameter. |
| ***Aspergillus tamarii*** | Dark brown colonies, 6 cm on CzA and 7 cm on MEA after 13 days at 25 °C. | Conidiophores hyaline, 1–2 mm long, rough, with globose vesicles (25–50 µm) and sterigmata in one or two series. | Conidia cylindrical when immature, becoming globose at maturity, 5–6 µm in diameter. |
| ***Aspergillus terreus*** | Buff to cinnamon colonies, floccose with yellow reverse, 5.2 cm diameter in 10 days at 25 °C on CzA. | Conidiophores hyaline, smooth-walled; conidial heads compact, biseriate, columnar, 0.5 mm long, 30–50 µm wide. | Conidia hyaline to pale yellow, globose, smooth, 1.6–2.4 µm in diameter. |
| ***Aureobasidium pullulans*** | Pinkish colonies on PDA, smooth, slimy, rapidly expanding, later becoming black due to chlamydospore formation; 2.9–4.65 cm on MEA after 7 days. Endoconidia commonly produced within yeast-like cells. | | |
| ***Bipolaris hawaiiensis*** | Rapidly growing colonies, white at first, turning dark gray to black with woolly to cottony texture at 25 °C. | Conidiophores sympodial, geniculate, simple or branched, up to 110 µm long, bearing conidia via pores. | Conidia oblong to cylindrical, pale to medium brown, 2–6 distoseptate, 12–35 × 5–10 µm, nonprotuberant hilum, germinating bipolarly. |
| ***Bipolaris maydis*** | Greyish to dark green-black cottony colonies with irregular margins, 45–75 mm after 14 days at 25 °C, reverse black. | Conidiophores septate, straight or slightly curved, producing germ tubes at both ends. | Conidia dark brown, fusiform, thick-walled, 4–10 septate, 65–87 × 14–16 µm. |
| ***Bipolaris tetramera*** | Brownish, glassy colonies covered with conidial powder, 3 cm after 5 days at 25 °C on OA. | Conidiophores brown, unbranched, septate, 250 × 4–8 µm, single or clustered (2–3). | Conidia brown, ellipsoidal, straight with rounded ends, lighter at tips, 20.4–35.7 × 8.5–13.6 µm, 3-septate. |
| ***Botrytis cinerea*** | White to pale gray, fluffy colonies with dense mycelium, growing 27 mm/day at 25 °C on PDA. | Conidiophores upright, hyaline, thin-walled, sparsely septate, apically branched, turning brown when mature. | Conidia single-celled, pyriform to globose, smooth, 7.2–12.4 × 5.1–9.5 µm; sclerotia irregular, gray to black, 2–4.6 mm. |
| ***Cephalosporium acremonium*** | Pinkish colonies with central tufts, 1.8–2.3 cm after 10 days at 22 °C on MEA. | Simple, thin-walled phialides arising from submerged hyphae. | Cylindrical conidia (3.5 × 1.2 µm); chlamydospores with pigmented walls (4–8 µm) form after 12 days. |
| ***Chaetomium* spp.** | Cottony colonies, white to olive-gray, 7 cm in 10 days at 20 °C on OA. | Dark brown to black perithecia with large cylindrical asci. | Brown, lemon-shaped, unicellular ascospores. |
| ***Cladosporium* spp.** | Slow-growing, olivaceous-green colonies with velvety to powdery texture on PDA at 25 °C. | Conidiophores tall, dark, upright, variably branched near apex, either clustered or solitary. | Conidia dark, ovoid, 1–2-celled, irregular, arranged in simple or branched chains. |
| ***Colletotrichum* spp.** | Olive-green to gray slimy colonies, 5 cm after 5 days at 25 °C on PDA. | Acervuli scattered, 100–250 µm across, bearing short conidiophores (8–10 × 2–4 µm). | Conidia cylindrical, hyaline, straight, nonseptate, rounded ends, 11–13 × 3–4 µm. |
| ***Curvularia lunata*** | Velvety dark brown colonies, 6 cm after 5 days at 25 °C on OA. | Conidiophores straight, simple or branched, solitary or grouped, about 650 × 9 µm. | Conidia curved at the third cell, 3-septate, smooth-walled, 20 × 10 µm, middle cell larger and darker. |
| ***Epicoccum purpurascens*** | Yellow to brown colonies, 6 cm after 10 days at 20 °C on CzA or MEA. | Conidiophores hyaline, club-shaped, smooth, 0–2 septa, 9 × 6 µm, bearing one dark conidium terminally. | Conidia globose, 15–25 µm, golden to dark brown, funnel-based, divided into up to 15 cells. |
| ***Fusarium incarnatum*** | Whitish colonies turning buff-brown, 6 cm in 4 days at 25 °C on OA. | Conidiophores branched, bearing slender cylindrical phialides (19–24 × 2–4 µm), each forming one conidium. | Macroconidia fusiform, straight with beaked tips, 3–5 septa, 17–35 × 3–4 µm; chlamydospores globose, 5–10 µm. |
| ***Fusarium verticillioides*** | Whitish to pale orange colonies, 4 cm in 4 days at 25 °C on PDA. | Conidiophores of medium length, simple or branched, with monophialidic conidiogenous cells. | Macroconidia straight, 5-septate, 31–58 × 2.7–3.6 µm; microconidia abundant, 0–1 septate, oval to clavate, 7–10 × 2.5–3.2 µm. |
| ***Mucor* spp.** | White colonies turning dark gray, 9 cm after 4 days at 25 °C on MEA. | Conidiophores hyaline, lacking basal rhizoids, bearing large columellae and terminal sporangia. | Sporangiospores globose to ellipsoidal, hyaline to pale brown, smooth-walled. |
| ***Nigrospora* spp.** | Floccose white-gray colonies, reverse pale yellow-gray with black spots, 90 mm in 5 days at 25 °C. | Septate hyphae (2–4.5 µm); monoblastic, straight or flexuous conidiophores; ampulliform cells (8.2 × 6.3 µm). | Conidia black, solitary, spherical to ellipsoidal, 10.9–15.2 µm or 12–15.2 × 9–12 µm. |
| ***Penicillium* spp.** | Rapidly growing colonies, velvety, initially white, later gray-green on PDA. | Conidiophores branched near apex in penicillate structures ending in phialides. | Conidia hyaline, single-celled, globose to ovoid, forming chains. |
| ***Phoma* spp.** | Gray colonies, gradually darkening, 5.5 cm after 7 days at 25 °C on OA. | Pycnidia irregularly scattered across the colony surface. | Conidia ellipsoidal, two-celled (6 × 3 µm), with dark chlamydospores (10–22 µm). |
| ***Rhizoctonia solani*** | Brownish colonies with septate, multinucleate hyphae showing dolipore septa; branches arise at right angles, no spores formed. | | |
| ***Rhizopus stolonifera*** | Reddish gray-brown colonies, fast-growing, exceeding 2 cm high on PDA. | Conidiophores straight, pale to dark brown (2.5 × 20 µm), linked by hyaline to brown stolons with branched rhizoids. | Sporangia black (100–200 µm) with oval columellae; sporangiospores subglobose, ridged, 7 × 4.5 µm. |
| ***Stemphylium* spp.** | Colonies with hyaline to brown hyphae forming pulvinate stromata, 6–8 cm in 8 days at 25 °C on MEA. | Conidiophores straight, simple, 20–72 × 4–6 µm, dark brown, roughened apex. | Conidia ovoid, 2–3 septa, olive-brown, 24–38 × 15–26 µm. |
| ***Trichoderma* spp.** | Rapid-growing colonies on PDA, white initially, later yellow-green to deep green in concentric tufts, cottony to velvety texture. | Conidiophores repeatedly branched, dendritic; phialides flask-shaped, solitary or in whorls of 3–5, diverging at right angles. | Conidia green, ellipsoidal, smooth to rough, formed in slimy masses (gloiospora). |
| ***Ulocladium* spp.** | Black colonies with white margin on CMA; mycelium pale olive-brown, septate, 5 µm thick. | Conidiophores dark brown, erect, simple or branched, 5–8 × 120 µm. | Conidia solitary, dark brown, variable in shape, with one to three transverse septa. |

**Table S2. Colony-forming unit (CFU) counts of seed-borne endophytic fungi isolated from maize grains collected across 18 Egyptian governorates.**

| **Governorate** | **Ab. spp.** | **A. alt** | **Arth. spp.** | **Asp. clav** | **Asp. flav** | **Asp. fum** | **Asp. glau** | **Asp. Nig** | **Asp. Ochr** | **Asp. Tam** | **Asp. Terr** | **Auro. pull.** | **B. haw** | **B. may** | **B. tet** | **Bot. cin** | **Ceph.acr.** | **Chaet spp.** | **Cl. spp.** | **Coll. spp.** | **Cu. haw** | **Epi. Pur** | **F. inc** | **F. spp** | **Fus. Ver** | **Muc. spp.** | **Nig. spp.** | **Pen. spp.** | **Phom. spp.** | **R. so** | **R. sto** | **St. spp.** | **Trichod. spp.** | | **Ulo. spp.** | **Total CFU** |
| --- | --- | --- | --- | --- | --- | --- | --- | --- | --- | --- | --- | --- | --- | --- | --- | --- | --- | --- | --- | --- | --- | --- | --- | --- | --- | --- | --- | --- | --- | --- | --- | --- | --- | --- | --- | --- |
| **New Valley** | **0** | **13.9** | **0** | **0** | **74** | **4** | **0** | **228** | **0** | **0** | **4** | **94** | **0** | **12** | **0** | **0** | **0** | **0** | **16** | **0** | **0** | **2** | **0** | **0** | **16** | **0** | **13.9** | **124** | **0** | **0** | **0** | **0** | **4** | | **0** | **605.8** |
| **Luxor** | **2** | **0** | **0** | **4** | **264** | **1** | **1** | **151** | **4** | **0** | **3** | **156** | **0** | **4** | **0** | **0** | **2** | **2** | **56** | **0** | **0** | **3** | **9** | **3** | **62** | **0** | **36** | **106.2** | **0** | **0** | **7** | **10** | **37** | | **0** | **923.2** |
| **Assiut** | **0** | **18** | **0** | **1** | **88** | **8** | **26** | **46** | **0** | **0** | **4** | **216** | **0** | **2** | **0** | **0** | **0** | **4** | **118** | **0** | **0** | **4** | **8** | **0** | **36** | **0** | **22** | **110** | **0** | **0** | **0** | **4** | **12** | | **0** | **727.0** |
| **Aswan** | **0** | **1.6** | **0** | **6.4** | **168** | **0** | **0** | **171.2** | **1.6** | **0** | **38.4** | **12.8** | **0** | **1.6** | **0** | **1.6** | **0** | **0** | **0** | **0** | **0** | **0** | **0** | **0** | **81.6** | **0** | **1.6** | **41.6** | **0** | **0** | **3** | **0** | **45.2** | | **0** | **576.2** |
| **Al-Behera** | **0** | **6.7** | **0** | **2** | **25.3** | **41.3** | **2.7** | **17.3** | **1.3** | **0** | **0** | **36** | **2** | **0** | **1.3** | **0** | **0** | **10.7** | **40** | **0** | **0** | **0** | **16** | **8** | **61.3** | **4** | **8** | **69.3** | **1.3** | **0** | **0** | **4** | **2.7** | | **1.3** | **362.7** |
| **Al-Gharbia** | **0** | **2** | **0** | **0** | **136** | **0** | **0** | **50** | **0** | **0** | **4** | **113** | **0** | **0** | **0** | **0** | **8** | **4** | **4** | **0** | **0** | **0** | **0** | **12** | **132** | **0** | **2** | **64** | **0** | **0** | **0** | **2** | **24** | | **0** | **557.0** |
| **Al-Sharkia** | **0** | **0** | **0** | **0** | **18** | **0** | **0** | **16** | **0** | **0** | **0** | **0** | **0** | **0** | **0** | **0** | **18** | **0** | **0** | **1** | **0** | **0** | **0** | **20** | **68** | **0** | **0** | **18** | **0** | **0** | **26** | **0** | **12** | | **0** | **197.0** |
| **Al-Dakhlia** | **0** | **4** | **0** | **2** | **116** | **0** | **0** | **74** | **0** | **0** | **0** | **54** | **2** | **2** | **0** | **0** | **18** | **0** | **0** | **0** | **0** | **0** | **3** | **0** | **214** | **0** | **4** | **73.3** | **0** | **0** | **4** | **0** | **2** | | **0** | **572.3** |
| **Damietta** | **0** | **2.6** | **0** | **1.6** | **76.8** | **0** | **0** | **9.6** | **0** | **0** | **0** | **11.2** | **0** | **0** | **0** | **0** | **0** | **0** | **11.2** | **0** | **0** | **0** | **0** | **0** | **6.4** | **0** | **0** | **17.6** | **0** | **0** | **0** | **0** | **0** | | **0** | **137.0** |
| **Kafr El-Sheikh** | **0** | **0** | **0** | **0** | **22.4** | **1.6** | **0** | **241.6** | **0** | **0** | **0** | **0** | **0** | **1.6** | **0** | **0** | **0** | **6.4** | **1.6** | **0** | **0** | **0** | **0** | **0** | **652.8** | **0** | **3.2** | **68.8** | **0** | **0** | **1.6** | **0** | **8** | | **0** | **1009.6** |
| **Alexandria** | **0** | **8** | **0** | **0** | **40** | **0** | **0** | **108** | **0** | **0** | **28** | **186** | **0** | **6** | **0** | **0** | **6** | **10** | **18** | **0** | **0** | **0** | **6** | **0** | **26** | **0** | **2** | **80** | **0** | **0** | **0** | **0** | **4** | | **0** | **528.0** |
| **Al-Nobaria** | **0** | **13** | **0** | **0** | **281** | **2** | **2** | **366** | **0** | **0** | **0** | **49** | **0** | **2** | **0** | **0** | **1** | **2** | **51** | **0** | **1** | **2** | **1** | **0** | **17** | **0** | **40** | **104** | **0** | **0** | **0** | **0** | **2** | | **0** | **936.0** |
| **Al-Giza** | **0** | **6** | **0** | **2** | **88.2** | **0** | **0** | **39** | **2** | **0** | **0** | **352** | **0** | **2** | **0** | **0** | **0** | **22** | **14** | **0** | **0** | **2** | **0** | **4** | **18** | **2** | **28** | **516** | **0** | **0** | **0** | **0** | **14** | | **0** | **1111.2** |
| **Al-Fayoum** | **0** | **0** | **0** | **0** | **21** | **6** | **0** | **167** | **0** | **1** | **0** | **0** | **0** | **0** | **0** | **3** | **51** | **0** | **11** | **0** | **0** | **1** | **0** | **3** | **94** | **0** | **11.4** | **331** | **0** | **0** | **0** | **0** | **2** | | **0** | **702.4** |
| **Al-Ismaelia** | **0** | **22** | **0** | **0** | **96** | **0** | **0** | **80** | **0** | **0** | **34** | **310** | **0** | **8** | **0** | **4** | **2** | **0** | **36** | **0** | **4** | **4** | **14** | **14** | **66** | **0** | **28** | **270.0** | **0** | **0** | **6** | **0** | **28** | | **0** | **1026.0** |
| **Port Said** | **0** | **4.8** | **0** | **102.4** | **96** | **0** | **0** | **256** | **0** | **0** | **0** | **76.8** | **0** | **8** | **0** | **6.4** | **9.6** | **3.2** | **108.8** | **0** | **0** | **4.8** | **0** | **8** | **161.6** | **0** | **3.2** | **77.2** | **0** | **0** | **3.2** | **0** | **0** | | **0** | **930.0** |
| **Al-Menia** | **1.3** | **9.3** | **170.7** | **2.7** | **25.3** | **0** | **0** | **68** | **0** | **0** | **17.3** | **0** | **0** | **26.7** | **0** | **0** | **2.7** | **2.7** | **66.7** | **0** | **2.7** | **0** | **5.7** | **1.3** | **12** | **2.7** | **14.7** | **89.3** | **0** | **0** | **0** | **13.3** | **26.7** | | **0** | **561.7** |
| **North and South Sinai** | **0** | **14.7** | **0** | **2.7** | **48** | **0** | **4** | **130.7** | **0** | **0** | **0** | **0** | **0** | **0** | **0** | **0** | **17** | **8** | **90.7** | **0** | **0** | **1.3** | **8** | **2.7** | **100** | **8** | **6.7** | **294.7** | **0** | **1.3** | **6.7** | **12** | **5.3** | | **0** | **762.3** |
| **Egypt** | **3.3** | **126.5** | **170.7** | **126.7** | **1684.1** | **63.9** | **35.7** | **2219.4** | **8.9** | **1** | **132.7** | **1666.8** | **4** | **75.9** | **1.3** | **15** | **135. 3** | **74.9** | **642.9** | **1** | **7.7** | **24.1** | **70.7** | **76** | **1824.7** | **16.7** | **224.6** | **2455.1** | **1.3** | **1.3** | **57.5** | **45.3** | | **228.9** | **1.3** | **12225.4** |

Total Colonies per Governorate were calculated by summing all CFU values across identified fungal species in each governorate. The final row “Egypt” represents the total CFUs across all sampled governorates. *Ab*. spp. (*Absidia* spp.), *A. alt*. (*Alternaria alternata*), *Arth.* spp. (*Arthrobotrys* spp.), *Asp. cla*. (*Aspergillus clavatus*), *Asp. fla.* (*A. flavus*), *Asp. fum*. (*A. fumigatus*), *Asp. gla*. (*A. glaucus*), *Asp. nig*. (*A. niger*), *Asp. och*. (*A. ochraceus*), *Asp. tam.* (*A. tamarii*), *Asp. terr.* (*A. terreus*), *Auro. pul.* (*Aureobasidium pullulans*), *B. haw*. (*Bipolaris hawaiiensis*), *B. may.* (*B. maydis*), *B. tet.* (*B. tetramera*), *Bot. cin.* (*Botrytis cinerea*), *Ceph. acr.* (*Cephalosporium acremonium*), *Chaet.* spp. (*Chaetomium* spp.), *Cl.* spp. (*Cladosporium* spp.), *Coll.* spp. (*Colletotrichum* spp.), *Cu. lun*. (*Curvularia lunata*), *Epi. pur.* (*Epicoccum purpurascens*), *F. inc.* (*Fusarium incarnatum*), *Fus. spp.* (*Fusarium* spp.), *F. ver.* (*F. verticillioides*), Muc. spp. (*Mucor* spp.), Nig. spp. (*Nigrospora* spp.), Pen. spp. (*Penicillium* spp.), Phom. sp. (*Phoma* sp.), *R. so.* (*Rhizoctonia solani*), *R. sto.* (*Rhizopus stolonifer*), *St.* spp. (*Stemphylium* spp.), *Trichod. spp.* (*Trichoderma* spp.), and *Ulo*. spp. (*Ulocladium* spp.). The final row labeled “Egypt” in the table represents the mean frequency (%) of each fungal species across all surveyed governorates, providing an overall profile of seed-borne fungal prevalence. This summary row was excluded from comparative analyses and visualization.

**Table S3**. Total number of fungal colonies, species richness, and Shannon–Wiener diversity index (H) per governorate, along with mean ± SD of diversity indices based on eight site-level samples per region.

| **Governorate** | **Total colonies per governorate** | **Richness**  **per governorate** | **H index (Collected)** | **Richness ± SD**  **(Mean per site)** | **H index ± SD**  **(Mean per site)** |
| --- | --- | --- | --- | --- | --- |
| **New Valley** | 605.8 | 13 | 1.80 | 11.00 ± 2.33 | 1.72 ± 0.17 |
| **Luxor** | 923.2 | 22 | 2.12 | 11.13 ± 1.25 | 1.74 ± 0.32 |
| **Assiut** | 727.0 | 18 | 2.14 | 14.13 ± 3.31 | 1.98 ± 0.25 |
| **Aswan** | 576.2 | 14 | 1.81 | 9.13 ± 3.40 | 1.56 ± 0.21 |
| **Al-Behera** | 362.7 | 22 | 2.46 | 12.75 ± 5.06 | 2.11 ± 0.30 |
| **Al-Gharbia** | 557.0 | 14 | 1.92 | 10.88 ± 3.48 | 1.77 ± 0.23 |
| **Al-Sharkia** | 197.0 | 9 | 1.92 | 7.00 ± 1.60 | 1.74 ± 0.26 |
| **Al-Dakhlia** | 572.3 | 14 | 1.76 | 9.75 ± 2.66 | 1.60 ± 0.22 |
| **Damietta** | 137.0 | 8 | 1.45 | 5.50 ± 2.83 | 1.09 ± 0.63 |
| **Kafr El-Sheikh** | 1009.6 | 11 | 1.02 | 7.50 ± 3.02 | 0.77 ± 0.37 |
| **Alexandria** | 528.0 | 14 | 1.94 | 10.75 ± 3.73 | 1.71 ± 0.40 |
| **Al-Nobaria** | 936.0 | 17 | 1.65 | 8.50 ± 2.27 | 1.65 ± 0.42 |
| **Al-Giza** | 1111.2 | 16 | 1.49 | 12.50 ± 3.93 | 1.44 ± 0.20 |
| **Al-Fayoum** | 702.4 | 13 | 1.52 | 7.00 ± 1.51 | 1.38 ± 0.20 |
| **Al-Ismaelia** | 1026.0 | 18 | 2.08 | 15.25 ± 3.15 | 2.00 ± 0.20 |
| **Port Said** | 930.0 | 16 | 2.08 | 12.63 ± 3.29 | 1.84 ± 0.37 |
| **Al-Menia** | 561.7 | 20 | 2.24 | 11.00 ± 6.32 | 1.65 ±0 .73 |
| **North and South Sinai** | 762.3 | 19 | 1.94 | 11.00 ± 4.93 | 1.62 ± 0.41 |

**Table S4.** Antagonistic activity of different *Trichoderma* isolates against *Fusarium verticillioides* in dual culture assays, illustrating comparative inhibition rates used to select the most effective biocontrol candidate

| ***Trichoderma* isolates** | **Growth of pathogens (%)^1^** | |
| --- | --- | --- |
|  | **Growth inhibition (%)^1^** | **Antagonism reaction ^2^** |
| T14+F | 74.03 ± 2.1 ^a3^ | 1 |
| T30+F | 73.48 ± 1.84^ab^ | 1 |
| T34+F | 71.67 ± 2.13^a-c^ | 1 |
| T16+F | 71.11 ± 2.57^a-c^ | 1 |
| T5+F | 70.16 ± 2.97^a-d^ | 2 |
| T40+F | 69.44 ± 1.11^a-e^ | 2 |
| T8 +F | 69.07 ± 1.53^a-e^ | 1 |
| T6 +F | 69.05 ± 2.08^a-e^ | 1 |
| T13+F | 68.5 ± 1.26^a-e^ | 1 |
| T10+F | 68.49 ± 2.41^a-e^ | 1 |
| T23+F | 68.33 ± 1.11^a-e^ | 1 |
| T27+ F | 67.95 ± 1.5^a-e^ | 1 |
| T37+F | 67.78 ± 1.28^a-e^ | 2 |
| T9+F | 67.4 ± 2.1^a-f^ | 2 |
| T29+F | 67.4 ± 1.05^a-f^ | 1 |
| T 87+F | 67.22 ± 2.13^a-f^ | 1 |
| T 82+F | 67.22 ± 1.11^a-f^ | 1 |
| T11+F | 66.85 ± 1.85^a-g^ | 1 |
| T60+F | 66.85 ± 1.85^a-g^ | 2 |
| T102 | 66.84 ± 2.78^a-g^ | 1 |
| T78+F | 66.32 ± 3.02^b-g^ | 2 |
| T68+F | 66.3 ± 1.95^b-g^ | 2 |
| T50+F | 66.3 ± 0.72^b-g^ | 1 |
| T7+F | 66.01 ± 3.4^c-g^ | 1 |
| T103+F | 65.75 ± 1.11^c-g^ | 1 |
| T38+F | 65.75 ± 1.11^c-g^ | 2 |
| T4 +F | 65.74 ± 2.37^c-g^ | 2 |
| T52+F | 65.74 ± 1.52^c-g^ | 1 |
| T81+F | 65.56 ± 1.28^c-g^ | 2 |
| T101 | 65.17 ± 3.11^c-g^ | 1 |
| T25+F | 64.65 ± 1.5^c-h^ | 1 |
| T26+F | 64.64 ± 1.86^c-h^ | 1 |
| T104 | 64.64 ± 0.39^c-h^ | 1 |
| T 24+F | 64.44 ± 1.81^c-h^ | 1 |
| T76+F | 63.53 ± 1.54^d-i^ | 1 |
| T18+F | 63.33 ± 1.28^d-i^ | 1 |
| T12 +F | 63.16 ± 4.54^d-i^ | 1 |
| T 80+F | 62.22 ± 1.81^e-j^ | 1 |
| T46+F | 60.24 ± 3.72^f-j^ | 2 |
| T51+F | 60.22 ± 3.65^f-j^ | 1 |
| T45+F | 60.22 ± 3.17^f-j^ | 1 |
| T49+F | 60.21 ± 2.82^f-j^ | 1 |
| T48+F | 59.67 ± 3.21^g-j^ | 1 |
| T 55+F | 59.66 ± 3.86^g-j^ | 1 |
| T36+F | 57.78 ± 2.57^h-k^ | 1 |
| T 79+F | 56.67 ± 1.28^i-l^ | 2 |
| T69+F | 55.24 ± 4.65^j-l^ | 2 |
| T2 +F | 50.76 ± 4.41^kl^ | 1 |
| T1 +F | 50.69 ± 5.8^klc^ | 1 |
| T3 +F | 49.65 ± 4.09^l^ | 1 |

^1^ Growth inhibition of F. verticillioides (%) = (Radius growth for F. verticillioides in the presence of *Trichoderma*) / (Radius of growth in the lack of *Trichoderma*) x 100*.* ^2^ The antagonism reactions of *Trichoderma* with F. verticillioides was recorded based on the antagonism scale of Bell *et al*. (1982) after the 5th day of dual growth, using a scale of 1 to 5, where: 1 = *Trichoderma* overgrowing F. verticillioides and 5 = F. verticillioides overgrowing *Trichoderma*.^3^ Means within a column followed by different letter(s) are significantly different according to Tukey’s HSD test at p≤0.05. Values are presented as mean ± standard deviation (n=4).

Table S5. BLASTn (nucleotide BLAST**)** alignment for the nucleotide sequence and the similarity matches to *Trichoderma longibrachiatum* (T14) (PP768163.1).

| **Species** | **Scientific name** | **Accession number** | **Percentage of homology (%)** |
| --- | --- | --- | --- |
| *Trichoderma longibrachiatum* strain 18EPIT002 translation | *Trichoderma longibrachiatum* | MT881871.1 | 100 |
| *Trichoderma longibrachiatum* isolate JG9-31 translation | *Trichoderma longibrachiatum* | MN195113.1 | 100 |
| *Trichoderma longibrachiatum* strain 10-5 translation elongation | *Trichoderma longibrachiatum* | KF267252.1 | 100 |
| *Trichoderma longibrachiatum* strain 18AMEL004 translation | *Trichoderma longibrachiatum* | MT671921.1 | 100 |
| *Trichoderma longibrachiatum* strain 18EPLE009 translation | *Trichoderma longibrachiatum* | MT881874.1 | 100 |
| *Trichoderma bissettii* partial tef1a gene | *Trichoderma bissettii* | HG931266.1 | 100 |
| *Trichoderma longibrachiatum* strain 18ELIB022 translation | *Trichoderma longibrachiatum* | MT881869.1 | 100 |
| *Trichoderma longibrachiatum* strain ALG01 translation elongation | *Trichoderma longibrachiatum* | MT472841.1 | 100 |
| *Trichoderma longibrachiatum* isolate T10 translation elongation | *Trichoderma longibrachiatum* | OK500004.1 | 100 |
| *Trichoderma longibrachiatum* strain 18EPAE005 translation | *Trichoderma longibrachiatum* | MT881870.1 | 100 |
